# Facile synthesis of pristine graphene-palladium nanocomposites with extraordinary catalytic activities using swollen liquid crystals

**DOI:** 10.1038/srep33053

**Published:** 2016-09-13

**Authors:** T. Vats, S. Dutt, R. Kumar, P. F. Siril

**Affiliations:** 1 School of Basic Sciences, Indian Institute of Technology Mandi, Himachal Pradesh, India

## Abstract

Amazing conductivity, perfect honeycomb sp^2^ arrangement and the high theoretical surface area make pristine graphene as one of the best materials suited for application as catalyst supports. Unfortunately, the low reactivity of the material makes the formation of nanocomposite with inorganic materials difficult. Here we report an easy approach to synthesize nanocomposites of pristine graphene with palladium (Pd-G) using swollen liquid crystals (SLCs) as a soft template. The SLC template gives the control to deposit very small Pd particles of uniform size on G as well as RGO. The synthesized nanocomposite (Pd-G) exhibited exceptionally better catalytic activity compared with Pd-RGO nanocomposite in the hydrogenation of nitrophenols and microwave assisted C-C coupling reactions. The catalytic activity of Pd-G nanocomposite during nitrophenol reduction reaction was sixteen times higher than Pd nanoparticles and more than double than Pd-RGO nanocomposite. The exceptionally high activity of pristine graphene supported catalysts in the organic reactions is explained on the basis of its better pi interacting property compared to partially reduced RGO. The Pd-G nanocomposite showed exceptional stability under the reaction conditions as it could be recycled upto a minimum of 15 cycles for the C-C coupling reactions without any loss in activity.

In the history of science, the year 2004 would be entitled as the birth year of the youngest member of the carbon family “The Graphene^1^”. Tougher than steel, stronger than diamond and yet the thinnest material in the entire universe^2^,^3^. The fascinating properties of graphene solely depend on its unique honeycomb arrangement of sp^2^ bonded carbon atoms in a 2-D monolayer sheet. Graphene shows lots of amazing properties due to this unique structural arrangement. Among the various properties, great conductivity, large surface area that theoretically exceeds 2000 m^2^/g and tunable surface properties makes graphene a perfect support material for the nano-catalysts^4^,^5^,^6^.

RGO is the most commonly used form of graphene as a catalyst support as well as in most other applications. It interacts with metal nanoparticles easily, could be synthesized in bulk and very much prone to modification. However RGO contains marginal amount of residual (~8 atomic %) oxygen that is bonded to approximately 20% of the carbon atoms in sp^3^ arrangement^7^. The presence of these sp^3^ sites disrupts the flow of charge carriers through sp^2^ clusters. It also decreases the pi electron cloud in sheets that leads to compromised pi -pi interaction property of RGO sheets with other unsaturated systems^8^,^9^. Such defects in the support materials affect the catalytic property of nano-composites. Pristine graphene on the other hand contains minimal defect concentration and uniform pi- electron distribution all along its 2-D sheets proving itself a better catalyst support without any compromised property when compared to RGO.

Unfortunately, pristine graphene as such is a chemically inert material with relatively low interacting affinity towards metal nanoparticles than RGO^10^. Interaction between graphene sheet and the metal nanoparticles has to be ensured in the graphene-metal nanocomposites so that the catalyst particles do not get detached from the support^11^. Additionally, uniform distribution of metal nanoparticles on the support also has to be ensured. Thus, electro-mechanical coupling between the nanoparticles and the graphene sheets has to be achieved. A number of different strategies are being used for preparing nanocomposites of graphene. These include, introducing vacancies, applying strain or functionalizing the graphene sheet^12^. Vacancies due to dangling bonds behave like traps for metal nanoparticles and give proper stability to the composites^13^ Other methods like introduction of oxy functional groups in the sheets give the best nucleation sites for the growth of metal nanoparticles^14^. It is evident that in each of these methods, a compromise has been made in the form of defected graphene structure. Hence, there is a need to develop novel methods for the preparation of nanocomposites of graphene, while preserving its pristine properties.

A unique and novel approach of using SLCs as templates for the preparation of nanocomposites using pristine graphene is presented in this paper. The same method can be used for the preparation of nanocomposites of RGO also. SLCs have already proved themselves as excellent templates for directing the structure of nanomaterials such as metals, polymers and nanocomposites^15^. In the present study, the exfoliated graphene was trapped along with a Pd-precursor in the self-assembly of infinitely long surfactant–stabilized oil tubes those are regularly arranged to form a hexagonal lattice in brine solution to prepare the nanocomposites. Pd nanoparticles got deposited over pristine graphene sheets on exposure of the SLCs to a reducing agent.

Pd has many industrial applications, especially in C-C coupling reactions as well as for the hydrogenation of polyunsaturated hydrocarbons. Here we have explored the catalytic activity of the nanocomposites of Pd with G and RGO in the hydrogenation of nitrophenols and in C-C coupling reactions. Nitrophenols are the most crucial starting materials for the synthesis of aminophenols and also listed among the top 114 organic pollutants by United State environmental protection agency (USEPA)^16^. Pd-based catalysts are known to be capable of selectively hydrogenating nitro-compounds to their corresponding amino compounds for the environmental remediation of nitrophenols^17^. The C-C bond formation between two hydrocarbon fragments with the help of metal catalysts has a very important place among different organic reactions. The comparative study showed the superior catalytic activity of Pd-G over Pd-RGO as well as many other Pd/graphene nanocomposites that are reported in the literature. Inclusion of graphene, more specifically pristine graphene in liquid crystals itself is a highly interesting and challenging task that has been achieved in this report^18^. The ordered arrangement of graphene in SLCs can be explored for making many other useful nanocomposites with metals, polymers, metal oxides and semiconductors.

## Results and Discussion

### Preparation and characterization of graphene

Sonication of graphite in surfactant solution is a reliable method for the synthesis of defect free graphene flakes^19^. The detailed synthesis process, its optimization and characterization of pristine graphene (G) and RGO are reported in electronic supporting information. UV-visible absorption spectra of graphene suspension in water are given in Figs S1 and S3 whereas the thermogravimetric analysis (TGA) thermal curves of G are given in Fig. S2. TEM imaging of G (Fig. S4) revealed the presence of thin graphene sheets. AFM imaging and statistical analysis given in Fig. S5 also confirmed the presence of thin graphene sheets. Raman spectroscopy (Fig. S6b) confirmed the relatively less defected structure of G when compared to RGO.

### Preparation and Characterization of SLCs

Transformation of the mixture of the aqueous phase containing surfactant and graphene and the oil phase containing the Pd salt from an opaque emulsion to a transparent and viscous gel (Fig. S7) gave the primary indication of the formation of hexagonal mesophase. Transparency of the SLCs is a clear confirmation of the complete dispersion of thin graphene sheets in them. The formation of SLCs was confirmed further by polarized optical microscopy imaging (Fig. S8a,b) and small angle X-ray scattering (SAXS). SAXS patterns of two mesophase samples were recorded at room temperature. In one of the samples, the mesophases were formed without the presence of graphene (SLC-Pd) whereas in the second sample, graphene was present in the mesophase (SLC-G1). The SAXS data for SLC-Pd and SLC-G1 are shown in supporting information as Fig. S8c,d respectively. The SAXS patterns of the samples showed three peaks, whose positions were in the ratio of 1:√3:2 and correspond respectively to (1 0), (1 1) and (2 0) diffraction planes of the hexagonal mesophases formed by the compact packing of cylindrical surfactant tubes^20^.

Formation of pristine graphene containing lyotropic liquid crystalline phases is reported for the first time here. Hence, it is interesting to know the locus of the graphene sheets in the mesophases. Self-assembly of surfactants on carbon nanotubes is well studied experimentally as well as theoretically^21^. However, experimental studies on the self-assembly of surfactants on graphene are relatively much less explored. Nevertheless, a number of theoretical studies are available on the subject. It has been proposed that hemi-spherical micelles of SDS are formed on graphene at critical micellar concentration (CMC). These micelles then get transformed into hemi-cylindrical when the concentration of SDS is increased much above CMC^22^. This has been experimentally proved through conductiometric studies on the adsorption of SDS on graphene^23^. Additionally, the hemi-spherical micelles that are present on the graphene surface can grow into hemi-cylindrical micelles when NaCl is added to the SDS/G aqueous suspensions^24^. A number of free cylindrical micelles may also form. The hemi-cylindrical micelles that are on the graphene sheets and the free cylindrical micelles further gain rigidity and hexagonal mesophases are formed when the required quantities of oil phase and co-surfactant were added.

**Figure 1:**
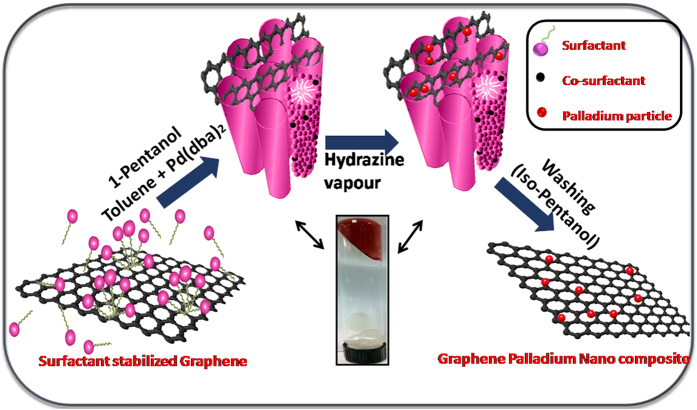
Graphical representation of the formation of a hexagonal mesophase containing graphene and the deposition of Pd nanoparticles on graphene on exposure to hydrazine vapour.

A graphical representation of the hexagonal mesophase is shown in Fig. 1. The hydrocarbon tail groups will be attached to the surface of graphene sheets^23^. This means that graphene sheets should be confined inside the oil phase of the SLCs. This seems quite unlikely as the diameter of the surfactant tubes were found to be 16.5 nm. The position of the first peak, q_10_ in the SAXS pattern of the mesophase, allows the direct determination of the center–center distance between adjacent tubes (d_c_) = (2/√3)(2п/q_10_)^20^. The d_c_ values for SLC-Pd and SLC-G1 were 16.35 and 16.5 nm respectively. Hence, large graphene sheets will have to roll up forming concentric tubes to be confined inside the surfactant cylinders. However, this is not possible as the lateral dimension of most graphene flakes was many micrometers. Thus, the graphene sheets must be extending out and cut across through many oil filled surfactant cylinders as shown graphically in the Fig. 1.

### Preparation and characterization of graphene palladium nanocomposite

**Figure 2:**
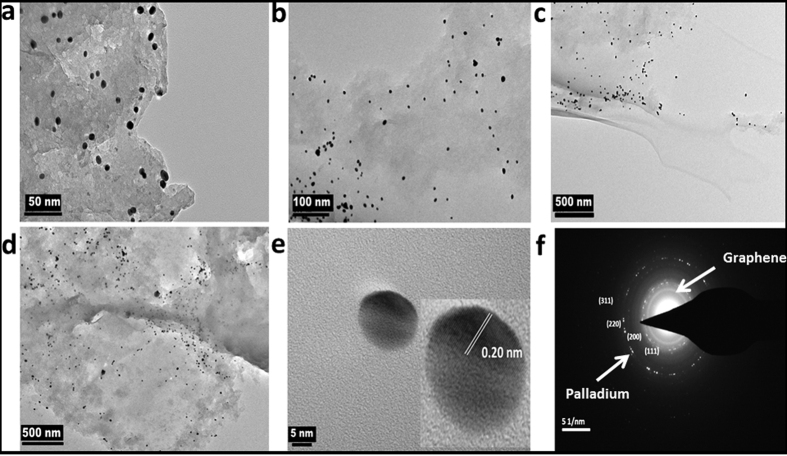
Characteristics of the Pd-G nanocomposite, GPd0.001M (**a**–**d**) TEM images showing the preferential deposition of spherical Pd nanoparticles on G, (**e**) high-resolution TEM image showing characteristic lattice fringes of Pd, (**f**) SAED pattern showing the fcc lattice planes of Pd and (1110) planes of graphene.

**Figure 3:**
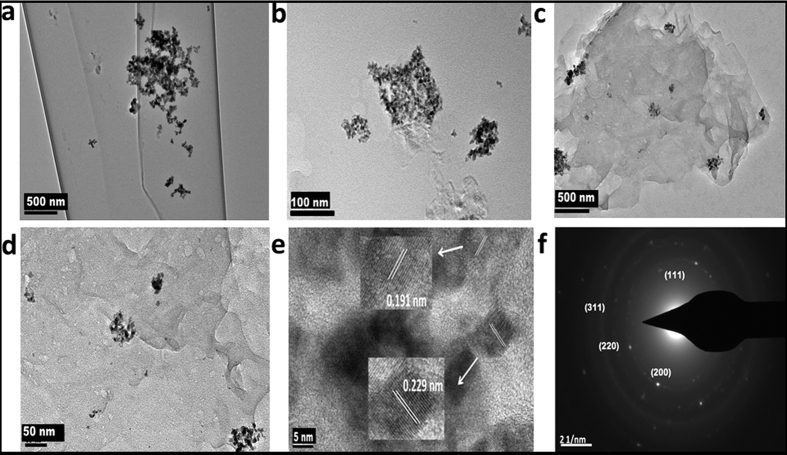
Characteristics of the Pd-G nanocomposite, GPd0.01 (**a**–**d**)TEM images showing the deposition of Pd nanoparticles on G, (**e**) high-resolution TEM image showing characteristic d-spacing of Pd, and (**f**) SAED pattern showing the characteristic fcc structure of Pd.

It is well known that dba is a quite labile ligand and hence it reacts quickly with reducing agents to yield Pd particles at ambient temperatures^25^. Exposing the SLC-Pd (SLC containing Pd(dba)_2_ without graphene) to hydrazine vapour yielded spherical nanoparticles of Pd (Fig. S9) in conformity with the previous reports^26^. Colour of all the mesophases, containing G or RGO also changed to black when they were exposed to hydrazine. This indicated the formation of Pd-nanoparticles. Formation of nanocomposites of graphene and spherical nanoparticles of Pd was confirmed by TEM imaging. Typical TEM images of the nano-composites, i.e. GPd0.001M and GPd0.01M that were prepared from SLCs containing the Pd(dba)2 dissolved in toluene with concentrations of 0.001 and 0.01 M respectively are shown in Figs 2 and 3. TEM image of RGPd0.001M (nanocomposite of RGO with Pd) is shown in Fig. S10 (ESI). Preferential deposition of Pd nanoparticles on graphene sheets can be seen in all the TEM images. Almost no particle was present outside the graphene sheets. This illustrates the efficient nanocomposite formation using the SLCs as templates.

The particles were mostly spherical in all the samples. A higher degree of agglomeration and overcrowding of Pd nanoparticles was seen in GPd0.01M than the other nanocomposite. This must be due to higher concentration of Pd salt (0.01 M) in the SLC from which the GPd0.01M was prepared. The average size of Pd nanoparticles calculated from TEM images and was 4.2 ± 1.6, 4.7 ± 1.4 and 7.7 ± 1.7 nm, respectively for GPd0.001M, RGPd0.001M and Pd0.001M. The narrow distribution of particle size in these nanocomposites is evident in the histograms those are shown in Fig. S11. From a comparison of the average particle sizes, it can be concluded that G and RGO were able to control the particle size, when they were present in the mesophases. They may be interfering in the mass transfer processes by acting as physical barriers. AFM images of GPd0.001 (Fig. S12) also show preferential deposition of Pd nanoparticles on graphene sheets.

**Figure 4:**
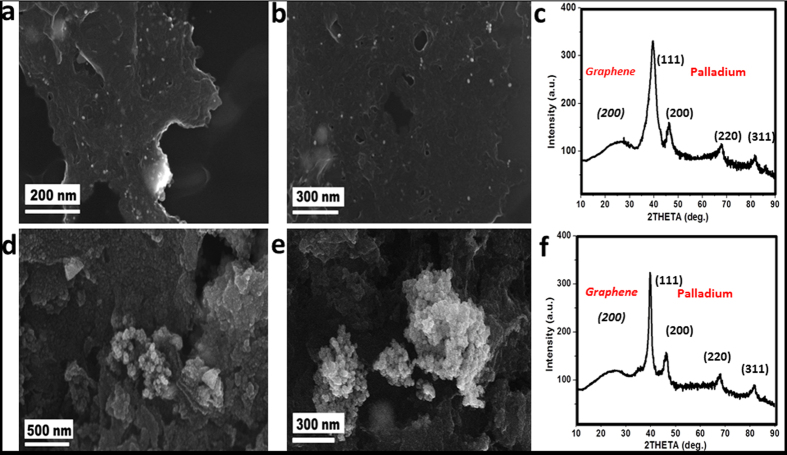
(**a**,**b**) FESEM images of GPd_0.001M_ showing the deposition of Pd nanoparticles on G, (**c**) XRD patterns showing the lattice planes of GPd_0.001M_. (**d**,**e**) FESEM images of GPd_0.01M_ and (**f**) XRD patterns showing the lattice planes of GPd_0.01M_.

The Pd nanoparticles were crystalline with fcc crystal structure according to the HRTEM images those are shown in Figs 2e and 3e respectively of GPd0.001M and GPd0.01M. Lattice spacing (d) value of 0.20 nm corresponding to (311) plane of Pd nanocrystals was observed for GPd0.001M, while d = 0.229 and 0.191 nm were observed for GPd0.01M corresponding to (111) and (311) planes of fcc Pd nanocrystals. The SAED patterns of the nanocomposites, GPd0.001M, GPd0.01M and RGPd0.001M are shown in Figs 2f and 3f and S10d respectively. All the nanocomposites exhibited similar SAED patterns. The bright spots corresponded to (111), (200), (220) and (311) planes of fcc Pd nanoparticles^27^,^28^. The XRD patterns of GPd0.001M and GPd0.01M are given in Fig. 4c,f respectively. The typical broad diffraction peak of exfoliated graphene was observed at 2θ = 24.8. The Four peaks corresponding to (111), (200), (220) and (311) planes of fcc crystal structure of Pd was also recorded in the XRD patterns^27^,^28^. Particle size of Pd nanoparticles in the nanocomposites was again reconfirmed by calculating the same from XRD data using Scherer formula. The particle size obtained from XRD data was 4, 4.2, 10.3 and 7.4 nm respectively for GPd0.001M, RGPd0.01M GPd0.01M and Pd0.001M.

**Figure 5:**
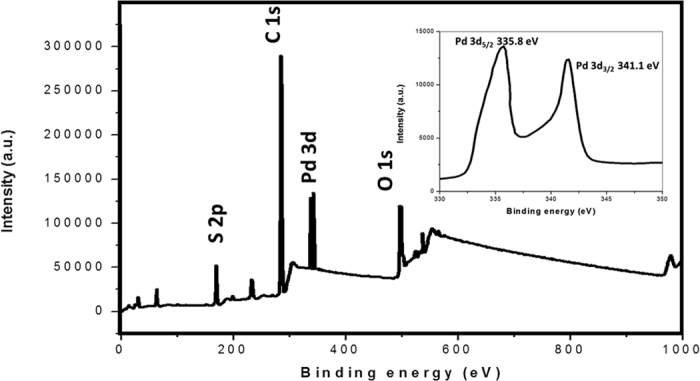
Overall XPS spectrum of the nanocomposite, GPd0.001 showing core levels of Pd, C and O. Inset figure shows Pd (0) 3d core-level XPS spectrum.

FESEM images for the nanocomposites, GPd_0.001M_ and GPd_0.01M_ are shown in Fig. 4. Very small, spherical particles of Pd, those are uniformly distributed on the graphene sheets can be seen in Fig. 4a,b corresponding to GPd_0.001M_. Aggregation of Pd nanoparticles can be seen in the FESEM images for GPd_0.01M_ (Fig. 4d,e). This is in agreement with the TEM data also. XPS characterization was done to study the surface elemental composition and oxidation state of GPd0.001. The overall XPS spectrum is shown in Fig. 5. High resolution XPS spectrum for the Pd 3d peak is shown in the inset of Fig. 5. The peaks corresponding to components of Pd 3d^5/2^ at 341.2 eV and Pd 3d^3/2^ at 335.9 eV are characteristic for Pd (0)^29^. However, the Pd 3d peak shape became asymmetric on the high binding energy side. Such peaks are usually attributed to the presence of small amount of oxides on the surface (PdO or PdO_2_)^29^.

Amount of Pd nanoparticles in the composites is one of the most important characteristics that determine their catalytic activity. Inductively coupled plasma mass spectrometry (ICP MS) helped in getting accurate concentration of Pd nanoparticles in synthesized composites. The amount of Pd in all the four composites were 70, 75, 82 and 98% by mass for GPd0.001M, RGPd 0.001M, GPd0.01M and Pd0.001M respectively.

### Particle growth mechanism and bonding between graphene and metal nanoparticles

Mostly, spherical particles of Pd were formed when the SLCs were exposed to hydrazine vapour. Formation of such spherical particles was observed previously when SLCs formed by SDS containing Pd_2_(dba)_3_ in the oil phase were exposed to hydrazine vapour^26^. Presence of graphene does affect the particle size. It has been concluded from the previous studies that hydrazine hydrogenates the unsaturated bonds of dba leading to its dissociation and formation of unstable Pd clusters. These clusters migrate to the interface and particle growth takes place at the oil-water interface^26^. It appears that, growth of Pd nanoparticles takes place on the surface of graphene in the SLCs. As discussed above, graphene sheets transcend across many surfactant cylinders and thus it passes through the aqueous phase in between the surfactant cylinders. The Pd nuclei must be diffusing out to the interface through the surface of graphene as they are unstable in the oil medium. They get anchored on the head group of SDS molecules those are adsorbed on the graphene with their tail groups orienting towards graphene.

A control reaction was done without forming the mesophase to check the importance of the hexagonal mesophase formation in controlling the particle size. Hydrazine vapours were passed through a mixture of graphene suspended in water, NaCl (0.1 M), SDS (0.4 g/ml) and solution of Pd(dba)_2_ in toluene (0.001 M). The composition of the mixture was same as that in the mesophase except the presence of the co-surfactant, which is critical for the formation of the mesophase. TEM image of the nanocomposite that was formed from the control sample is shown in Fig. S13. Pd nanoparticles having size in the range of 30 to 40 nm were observed in the TEM images. The particles were not preferentially deposited on graphene sheets. The bigger particle size of Pd nanoparticles and a non-preferential deposition of graphene sheets from the control sample clearly underline the importance of the SLC assembly in the formation of very small Pd nanoparticles that are preferentially deposited on graphene.

The bonding between the Pd nanoparticles and pristine graphene has already been studied^10^,^30^. The formation of dative bonds between the Pd and graphene sheets has been proved both experimentally and theoretically^31^. The participation of surfactant molecules that are adsorbed on graphene sheets acting as anchors also cannot be completely ruled out. Presence of some amount of residual surfactant was detected in GPd_0.001M_ nanocomposite using EDS spectroscopy (Fig. S14) and XPS. Very small amount of sulfur was detected in EDS spectra and this confirms the presence of a small amount of surfactant in the nanocomposite compared to Pd.

### Catalytic hydrogenation of nitrophenol

**Figure 6:**
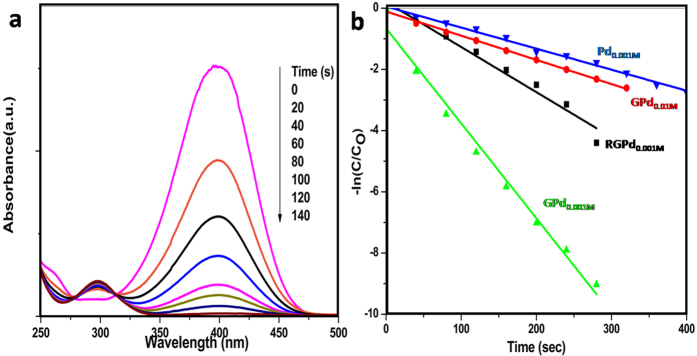
(**a**) Time-dependent UV-vis absorption spectra for the hydrogenation of p-NP using GPd0.001M as catalyst in aqueous medium by NaBH_4_ at room temperature and (**b**) Plot of -ln(Ct/C0) against reaction time for the catalytic hydrogenation of p-NP with different catalysts.

The catalytic efficiencies of all the four nanocomposites were compared by studying their activity in hydrogenation of p-NP. The progress of hydrogenation of p-NP was monitored by UV-visible absorption spectroscopy. Evolution of the UV-visible absorption spectrum of p-NP with time in presence of GPd0.001M as catalyst is shown Fig. 6a. The corresponding absorption spectra of p-NP using GPd0.01M, RGPd0.001M and Pd0.001M as catalysts are shown in Fig. S15. Progress of the reaction is clearly evident as there was decrease in the intensity of peak at 400 nm. There was concomitant increase in intensity of the peak at 300 nm corresponding to para-aminophenol with an isobestic point. On the addition of freshly prepared NaBH_4_, the absorption peak at 317 nm corresponding to p-NP shifted to 400 nm as shown in Fig. S16. The colour of the solution also changed from light yellow to bright yellow immediately. This red shift was due to the formation of p-nitrophenolate ions in alkaline conditions caused by the addition of NaBH_4_^32^.

The concentration of NaBH_4_ used was very much in excess (almost 100 times) compared to the concentration of p-NP. Hence, the reaction rate followed the pseudo first order kinetics with respect to the concentration of p-NP:


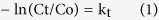


where, Ct and Co are the concentrations (mM) of the p-NP at time t and t0 respectively. The linear plots of –ln(Ct/Co) versus reduction time (t) for all the catalysts are shown in Fig. 6b. The observed rate constants are reported in Table 1 along with an arbitrary factor representing the catalytic efficiency of the metal (EoM) in the nanocomposites. EoM is defined as the number of moles of the product formed in unit time per moles of Pd in the catalyst. From the data, the order of activity of different catalysts is: GPd0.001M > RGPd0.001M > GPd0.01M > Pd0.001M.

It is interesting to note that the rate of p-NP reduction was more than twice higher in presence of GPd_0.001_ when compared with RGPd_0.001M_. More interestingly, the rate constant was more than 16 folds higher in presence of GPd_0.001_ than pure Pd_0.001M_. The better catalytic activity of GPd_0.001_ could be because of smaller particle size of Pd, good adsorption capacity and high conductivity of pristine graphene sheets than RGPd_0.001_ and Pd_0.001_. The uniform distribution of the small Pd nanoparticle also plays a key role as GPd_0.001_ showed much better catalytic activity than GPd_0.01_. A strong effect of particle size on the catalytic activity of various nano-materials for the reduction of NP was reported already^33^.

**Figure 7:**
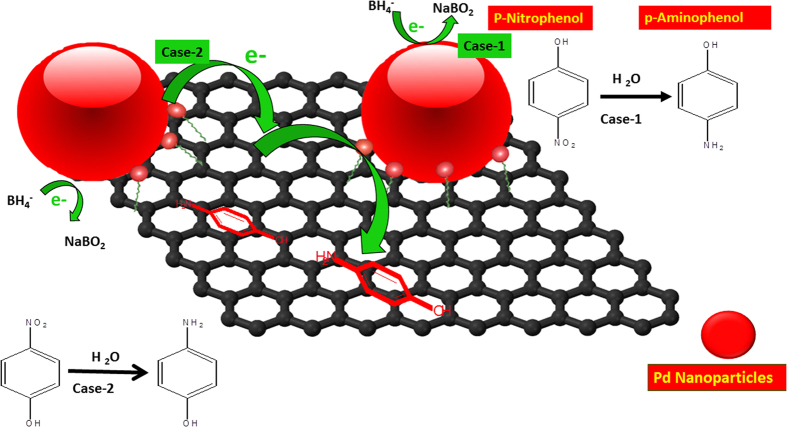
A plausible mechanism for the hydrogenation of p-nitrophenol catalyzed by the Pd/G nanocomposites in presence of sodium borohydride.

The hydrogenation reaction of solvated 4-nitrophenolate by BH^4−^ is quite a difficult reaction to proceed unmediated as the energy barrier between the two negative ions is quite high^34^. Thus, the presence of a catalyst (e.g., Pd NPs) is very much important for the completion of this redox reaction. According to the proposed mechanism in Fig. 7, the reaction starts by the supply of electrons from BH^4−^ ions to the catalyst and thereby allowing the p-NP absorbed on the catalyst to take electrons at their leisure^34^. It is already reported in the literature that, good π-π stacking interaction possibility of graphene makes it a very good adsorbent for the NPs^35^. Due to the enhanced adsorption of p-NP on graphene, the chances of hydrogenation are more through Pd NPs as the p-NP molecules can easily gain access to the Pd NPs. NaBH_4_ gets oxidized to BO^2−^when it comes in contact with the Pd NPs by transferring its electron to the Pd NPs. The electrons are then passed onto p-NP via graphene sheets or by direct contact with the metal particles. The better catalytic activity of GPd0.001 over RGPd0.001 could be explained on the basis of relatively low defect in the basal plane of G than RGO. Remember that the size of Pd particles and their concentration was almost the same in GPd0.001 over RGPd0.001. High defect concentration in RGO due to the presence of residual oxygen and distorted structure is well known^36^. This affects conductivity of electrons and the possibility for the adsorption of p-NP through π-π interaction. The better π-π interaction property of GPd0.001M over RGPd0.001M was proved by the better adsorption capacity of the former. Adsorption of p-NP on GPd_0.001_ and RGPd_0.001M_ was studied by following the change in concentration of p-NP solution using UV-visible absorption spectroscopy (Fig. S17). Adsorption capacity was 0.089 and 0.066 mg/g respectively for GPd_0.001M_ and RGPd_0.001M_. Better adsorption capacity of pristine graphene through π-π interaction thus makes it a better catalyst support compared to RGO.

As the GPd0.001M showed exceptional catalytic activity, the catalytic hydrogenation of o-NP and m-NP were also studied and the results are shown in Fig. S18a,b respectively. GPd0.001 was found to catalyse the different nitrophenols at varying rates. The reactivity was in the following order: m-NP > o-NP > p-NP. This difference in the reactivity is due to the difference in the stability of phenolate ions varying due to resonance and inductive effects. The stability of GPd0.001M was also studied by checking its recyclability in the p-NP reduction (Fig. S19). Good recyclability of the catalyst was established as GPd0.001M retained more than 85% of its original activity in the 5^th^ cycle. TEM image of the reclaimed catalyst after 5^th^ cycle (Fig. S20) showed continued attachment of the Pd NPs on the graphene support and established the stability of the catalyst. Thus, the small apparent loss of activity is attributed to the difficulty in efficiently reclaiming the very small amount of catalyst (0.24 mg) that was used for the reaction.

To verify the relative catalytic activity of our Pd-G nanocomposites, we compared their rate constants for p-NP hydrogenation reaction with other Pd based catalysts that are reported in the literature. The rate constant values are tabulated in Table S1 along with nature of the particles, size and concentration of the catalysts. A comparison of the rate constants reveals that GPd0.001 outperformed all other Pd-catalysts with different supports. The catalytic activity of GPd0.001 was many folds higher than the other Pd-based catalysts having similar or even smaller particle sizes. Better adsorption of p-NP through π-π interaction on pristine graphene and better conductivity of the catalyst support promoting efficient electron transfer could be the reasons for better catalytic activity.

### Catalytic Performance in C-C Coupling reactions under microwave irradiation

As the GPd0.001M showed exceptional catalytic activity in p-NP reduction, we have tested its catalytic activity in C-C coupling reactions as Pd is known to catalyze the same^37^. Catalytic activities were tested in three different coupling reactions, namely: Suzuki, Heck and Sonogashira reactions, under microwave irradiation. Detailed reaction schemes are presented in scheme S1 (supporting information). Detailed studies on optimization of reaction conditions for Suzuki coupling reaction, such as nature of base and solvent was done and the results are presented in Table S2 and S3 (supporting information). Best yields were obtained with K_2_CO_3_ as the base and a 1: 1 mixture of water and ethanol as solvent.

To compare the catalytic activity of prepared catalyst in coupling reaction, we performed the Suzuki coupling reaction between chlorobenzene and phenyl boronic acid using all the four different catalysts (GPd0.001, GPd0.01, RGPd0.001 and Pd0.001) and the results are presented in Table 2. GPd0.001 was the most active catalyst among all. The order of catalytic activity among the four catalysts in C-C coupling reaction was similar to the p-NP reaction. The better activity of GPd0.001 could be explained on the basis of graphene support and its amazingly perfect of π–π interacting property. Perfect π–π stacking interactions with the aromatic reactants helps in achieving the state of close proximity for the reactants and to the metal leading to increase in the rate of reactions^12^,^14^,^38^. Smaller size of the Pd nanoparticles in GPd0.001 should also be partially responsible for the better catalytic activity.

Catalytic activity of GPd0.001 was tested and established in various coupling reactions and the results are given in Table S4. GPd0.001 effectively catalyzes not only Suzuki reaction, but also Heck and Sonogashira C-C coupling reactions that too using a benign solvent mixture of water and ethanol. The Suzuki reaction took 45 min to complete. But the Heck and Sonogashira reactions completed within 10 min under microwave irradiation. More than 80% yield could be achieved in all the tested coupling reactions using chloro, bromo and iodo benzene as reactants. The catalytic activity of GPd0.001 for Suzuki coupling reaction was compared with other Pd and graphene nanocomposites that are reported in the literature. A careful comparison of the quantity of the catalyst, duration and the reactant used for the reaction revealed that the catalytic activity of GPd0.001 is superior to most of the other Pd/graphene nanocomposite catalysts. Comparable yields were reported using Pd/graphene nanocomposites in the literature by using either higher amount of catalyst or longer duration of reaction. Some reports claimed higher yields with comparable quantities of catalysts that were used in the present work. But they used either bromobenzene or iodobenzene as reactants, whereas we got better yields by using even chlorobenzene. Chlorobenzene is cheaper than bromo- or iodo-benzene, yet has very low reactivity^39^.

Industrial application of the heterogeneous catalysts warrants the material to be stable and recyclable. The recyclability of GPd0.001 was tested in all the three C-C coupling reactions upto 7 cycles. The product yields for the Suzuki, Heck and Sonogashira reactions after the 7^th^ cycle was 67, 84 and 87% respectively as mentioned in Table 3. We believe that the minor loss in activity is due to the difficulty in reclaiming the very small quantity of catalyst (0.5 mg) that was used in the reactions. In order to prove this point, recyclability was tested without reclamation and purification. Reactions were performed in cycles after charging fresh reactants to the same reaction vessel without removing the product and the catalyst. Upto 15, 18 and 19 cycles respectively for Suzuki, Heck and Sonogashira reactions could be completed without any change in product yield. Thus, a large amount of products could be achieved in a batch process using only a small amount of GPd0.001 without doing separation and purification after each batch. Thus, the GPd0.001 could be used as a valuable industrial catalyst for performing C-C coupling reactions.

## Materials and Methods

### Materials

Hydrazine hydrate (80%), 1-pentanol, sodium borohydride and sodium chloride were purchased from Merck. Graphite powder, Pd(dba)2, toluene, sodium dodecyl sulphate (SDS), ortho(o)-, meta(m)- and para(p)-nitrophenols were purchased from Sigma Aldrich and used as received. Whatmann Anodisc® 25 filter of 20 nm pore size with the filtration setup was purchased from Millipore. Ultra-pure water (18.2 MΩ-cm) from double stage water purifier (Elga pure lab option-R7) was used for all the experiments.

### Methods

#### Synthesis of graphene

The detailed synthesis procedure for pristine graphene and RGO are explained in supporting information.

#### Synthesis of the nanocomposites

The nanocomposites were made in two steps. The G or RGO was first entrapped in SLCs along with Pd(dba)2 and then exposed to hydrazine vapour to prepare the nanocomposites.

### Preparation of SLCs

G or RGO containing SLCs were prepared by modifying the reported procedure^28^,^29^. Typically, the dispersion of G in SDS (4 ml) was used to prepare 0.1 M NaCl solution. This solution was taken in a pyrex glass culture tube and then solution of Pd(dba)2 in toluene (6 ml, 1 × 10^−3 ^M) was vortex mixed with it. The co-surfactant (1-pentanol) was added in aliquots of 10 μl over a period of time to the above mixture with frequent vortex mixing to form the SLC. This SLC will be denoted as SLC-G1. A similar procedure was used to prepare the SLC with toluene containing a higher concentration (1 × 10^−2^ M) of Pd(dba)2. This will be called SLC-G2. An aqueous suspension of RGO (4 ml, 0.1 mg/ml) was used to prepare a solution containing SDS (0.4 g/ml) and NaCl (0.1 M). This was used to prepare the SLC by vortex mixing a solution of Pd(dba)2 in toluene (6 ml, 1 × 10^−3 ^M) and 1-pentanol (SLC-RG). An SLC containing Pd(dba) 2 (1 × 10^−3 ^M in toluene) without the presence of G or RGO was also prepared (SLC-Pd)^28^,^29^. The prepared mesophases were kept sealed and left to stabilize for few days before further studies.

### Synthesis of nanocomposites of Pd with G or RGO and Pd nanoparticles

Preparation of the nanocomposites and Pd nanoparticles was achieved by exposing the mesophases to hydrazine vapour for 72 h. Typically, a small amount of the mesophase was taken in a 10 ml glass vial. Then the vial was kept in a slightly bigger jar containing small amount of hydrazine. The bigger jar was kept sealed with glass cap and paraffin film. As hydrazine is toxic and carcinogenic, safety precautions like wearing gloves and mask was observed while handling them. Additionally, all the reactions involving hydrazine were conducted inside fume hood to avoid exposure. Synthesized nanomaterials were collected by dissolving the mesophases in iso-propyl alcohol. The samples were thoroughly washed by dispersing them in a mixture of isopropanol and water using ultrasonication for 10–15 min and then centrifuged at 3000 rpm to collect the residue. This process was repeated 8 times to remove the excess surfactants. Mesophases SLC-G1, SLC-G2, SLC-RG and SLC-Pd led to the formation of GPd0.001M, GPd0.01M, RGPd0.001M and Pd0.001M respectively.

#### Material characterization

UV-visible absorption spectra of graphene samples dispersed in water were recorded using Shimadzu UV-4250 and Perkin Elmer Lambda-750 spectrophotometers. Quartz cuvette that was used for the UV-visible spectroscopic characterization had an optical path length of 1 cm. TGA of filtered graphene was done using Netzcsh STA F1 Jupiter. Transmission electron microscopy (TEM) analyses were carried out using FEI Tecnai G2 200 S-Twin electron microscope, operating at 200 keV. FESEM imaging of the samples were carried out using FEI NOVA NANOSEM 450. AFM imaging of the graphene samples were done using Agilent SPM5500 in non-contact mode. The ICP-MS analysis of nanocomposites were done using Agilent 7900 instrument after digesting the samples in concentrated acid. Raman spectroscopy was carried out using RENISHAW in Via Raman spectrometer using a 532 nm laser at room temperature. XRD patterns of graphene and the nanocomposites were recorded using RIGAKU diffractometer at the 2θ angle range of 5° to 90° with CuKα radiation source (λ = 0.1542 nm, 40 mA, 45 kV). Crossed polarized optical microscopy (POM) imaging of the SLCs was done using Nikon ECLIPSE LV 100 POL optical microscope attached with a fire wire camera. A small amount of the mesophase was sandwiched between a clean glass slide and cover slip for POM imaging. Vacuum grease was applied at the edges of the cover slip to prevent the evaporation of the solvents. POM images of fresh mesophase were recorded immediately after deposition on the glass slides. Evolution of the liquid crystalline morphology with time was studied by recording POM images after 24 and 48 hours. SAXS patterns of SLCs were recorded using Rigaku Ultima IV fully automatic high resolution X-ray diffractometer system. Energy dispersive X-ray (EDS) spectra were recorded using a Bruker SDD detector attached with the TEM. The ^1^H and ^13^C spectra of the product of coupling reactions were recorded using JEOL JNM ECX 500 MHz NMR system in CDCl_3_.

#### Catalytic hydrogenation of nitrophenols

Catalytic hydrogenation reactions of nitrophenols were conducted at room temperature (25 °C) using the nanocomposites and the Pd-nanoparticles as catalysts. Typically, aqueous solutions of p-NP (3 mM) and NaBH4 (0.1 M) were freshly prepared. The catalyst (4 mg) was dispersed in 50 ml of p-NP solution with the help of ultra-sonication for several minutes. Then, a small portion (3 ml) of this mixture was taken in a quartz cuvette for studying the progress of reaction using UV-visible spectroscopy. The reaction was initiated by the addition of NaBH4 solution (0.1 ml) and the progress of the reaction was monitored by recording the evolution of absorption spectra with time. After the reaction, the catalysts were recovered by centrifugation and reused in succession without any further treatment. In addition, the hydrogenation of o-nitrophenol (o-NP) and m-nitrophenol (m-NP) was also carried out under the same conditions using the GPd0.001M, as the catalyst.

#### Catalytic Application in Carbon–Carbon Coupling Reactions

The efficiencies of catalysts in C-C coupling reactions were also tested. Three different coupling reactions; namely Suzuki, Heck and Sonogashira reactions were performed using microwave irradiation (Discover model, CEM).

### Suzuki reaction

Solvent (4 ml) was taken in a 10 ml reaction vessel. To this, halobenzene (1 eqv.), phenylboronic acid (1.2 eqv.), base (3 eqv.) and catalyst (0.5 mg) were added. The reaction vessel was then sealed and irradiated with microwaves for 45 min at 90 °C temperature.

### Heck reaction

Halo benzene (1 eqv.) was dissolved in the solvent mixture (4 ml) in microwave reaction vessel of 10 ml volume. Styrene (50 mg, 0.48 mmol, 2 eq.), potassium carbonate (99.7 mg, 0.72 mmol, 3 eq.) and the catalyst (0.5 mg) was added to reaction mixture. Then the tube was sealed and refluxed under microwave irradiation at 180 °C for 10 min.

### Sonogashira reaction

Sonogashira reaction was performed under similar experimental condition as the Heck reaction. Phenylacetylene (50 mg, 0.49 mmol, 2 eq.) was used instead of styrene as one of the reactants.

Progress of the reactions was monitored by TLC after performing many reactions of varying duration. After the completion of reaction, the reaction mixture was diluted with 20 ml water and extracted with 150 ml DCM (50 × 3 ml). The organic layer was separated and dried over anhydrous sodium sulphate and filtered. The solvent was removed using rotary evaporator under reduced pressure. The obtained solid was purified through column chromatography using silica gel column using hexane and ethyl acetate (9:1) solvent mixture before recording NMR spectra.

## Additional Information

**How to cite this article**: Vats, T. *et al*. Facile synthesis of pristine graphene-palladium nanocomposites with extraordinary catalytic activities using swollen liquid crystals. *Sci. Rep.* **6**, 33053; doi: 10.1038/srep33053 (2016).

## Supplementary Material

Supplementary Information

## Figures and Tables

**Table 1:** Comparison of catalytic activities of the Pd nanoparticle and the nanocomposites of Pd with graphene and RGO in p-nitrophenol hydrogenation reaction using NaBH4 as reducing agent.

S.No.	Catalyst	Rate constant (k)/s^−1^	k/k(Pd0.001M)	Efficiency of metal/s^−1^
1	GPd0.001M	0.133	16.6	1490.3
2	RGPd0.001M	0.059	7.4	1071.5
3	GPd0.01M	0.015	1.9	773.7
4	Pd0.001M	0.008	1	432.8

**Table 2:** Comparison of catalytic activities of the Pd nanoparticle and the nanocomposites of Pd with graphene and RGO in Suzuki-Miyaura coupling reaction between chlorobenze and phenyl boronic acid under microwave irradiation in 1:1 mixture of water and ethanol.

Catalyst	% Yield	Reaction time/h
GPd0.001	87	0.75
GPd0.01	73	0.75
RGPd0.001	78	0.75
Pd0.001	64	0.75

**Table 3:** Recyclability of GPd0.001M in Carbon–Carbon Cross Coupling reactions with iodo-benzene as one of the reactants under microwave irradiation in 1:1 mixture of water and ethanol.

Cycle	Suzuki (% yield)	Heck (% yield)	Sonogashira (% yield)
1	87	93	95
2	85	92	95
3	85	92	94
4	81	91	94
5	77	88	92
6	72	87	90
7	67	84	87
