# Supplementary material for: Facile synthesis of pristine graphene-palladium nanocomposites with extraordinary catalytic activities using swollen liquid crystals
Source: Sci Rep. 2016 Sep 13;6:33053. doi: 10.1038/srep33053 (PMC5020490; doi:10.1038/srep33053)
Supplement: Supplementary Information [file srep33053-s1.pdf]

# Supporting Information

## Facile synthesis of pristine graphene-palladium nanocomposites with extraordinary catalytic activities using swollen liquid crystals

Author: T. Vats, S. Dutt, R. Kumar and P.F. Siril

School of Basic Sciences, Indian Institute of Technology Mandi, Himachal Pradesh – 175 005, India

### Experimental details of the optimization of exfoliation conditions for the preparation of graphene:

Synthesis of pristine graphene

Exfoliated graphene was synthesized by modifying and optimizing the method reported by Loytaet. al.[1] Different parameters like concentration of surfactant, amount of graphite and duration of sonication were estimated to get the maximum concentration of graphene exfoliated in solution. The optimized conditions were used for preparing the pristine graphene. Typically, the solution of surfactant in water was prepared by dissolving SDS (0.4 g/ml) in a beaker. A desired dispersion of graphene was prepared by sonicating required amount of graphite (0.3 mg/ml) in the SDS solution for 6 h in a bath sonicator at room temperature. The resulting dispersion was left to stand for 24 h for letting the unstable and heavy aggregated flakes of graphene to settle down completely. The undisturbed solution was then centrifuged at 500 rpm for 90 min. The top half of the solution was pipetted out and retained for further use. The yield of graphene was calculated with the help of thermogravimetric analysis (TGA).

To get the maximum concentration of graphene exfoliated in solution different parameters like concentration of surfactant, amount of graphite and duration of sonication were studied in detail. For the exfoliation process, surfactant solutions of SDS in water were prepared by dissolving different concentration of surfactant in water (0.1, 0.2, 0.3 and 0.4 g/ml). Accurately weighed quantity of graphite powder (0.3 mg/ml) was then dispersed in the surfactant solutions. The suspension was then ultrasonicated in a bath sonicator for 6h. The resulting dispersion was left to stand for 24 h, for letting the unstable heavy aggregated flakes of graphene to settle down completely. The undisturbed solution was then centrifuged at 500 rpm for 90 min. The top half of the solution was pipetted out and retained for further use. UV-Visible spectra of all the exfoliated graphene solutions were recorded and are shown in, Fig. S1a. The optical density at 660 nm was used as an indicator of the concentration of graphene in the suspension.[2] From Fig. S1b, it is clearly evident that the maximum concentration of graphene was observed where the concentration of surfactant was 0.4 g/ml. However, the increase in concentration of graphene when the graphite concentration was raised from 0.3 mg/ml to 0.4 mg/ml was not significantly high. The optical absorption until 300 nm is predominated by SDS. UV-visible absorption spectra of aqueous SDS solutions having same concentrations as that were used for making graphene are shown in Fig. S1b. Comparison of Fig. S1a and S1b give a clear confirmation of broad range optical absorption

by graphene. Duration of sonication was found to be another important factor that affects the exfoliation. We found that concentration of graphene increased when sonication was done upto 6 h. There was no further increase in the concentration of graphene beyond 6 h of sonication as it shown in Fig. S1 c. Finally, it was concluded that best conditions for obtaining maximum graphene dispersion would be a combination of 0.4 g/ml surfactant solution, 0.3 mg/ml of graphite powder and 6 hours of sonication in a bath sonicator.

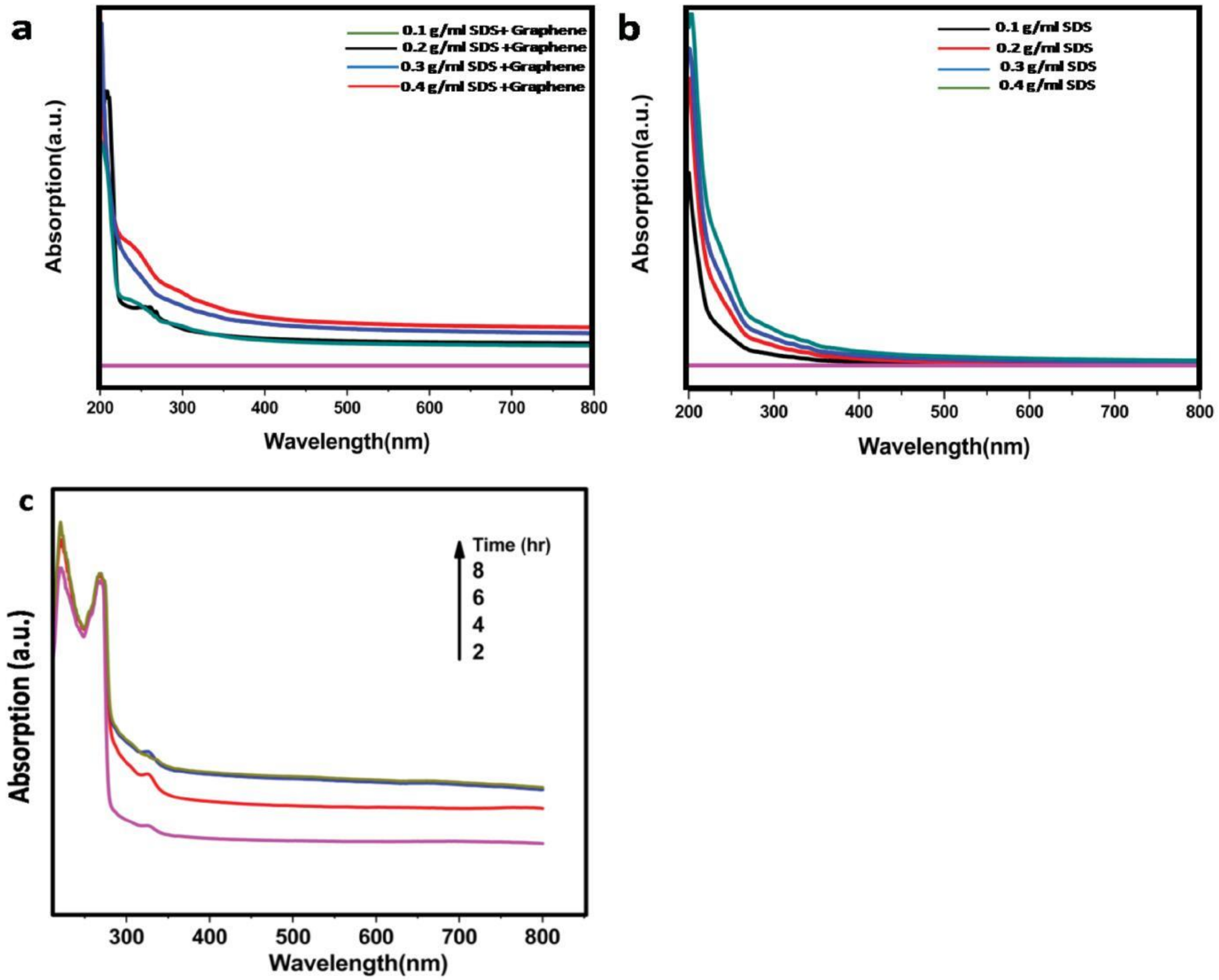


**Figure S1.** UV-Visible absorption spectra of (a) graphene dispersion in water that are stabilized by varying concentrations of SDS and (b) aqueous solution of varying concentrations of SDS and c) graphene dispersion in SDS solution at different sonication time.

## Finding out the yield of graphene

To calculate the yield of graphene synthesized using the optimized procedure, we prepared 400 ml of the graphene suspension. The solution was filtered through an inorganic filter membrane having pore size of 20 nm. The accurate mass of the filter membrane was recorded before filtration. Filtered graphene was washed thoroughly with double distilled water and vacuum dried in a hot air oven for 12 h to remove all traces of water. After weighing the dried graphene collected on the membrane, we could calculate the total mass of exfoliated graphene and residual SDS. Thermogravimetric analysis (TGA) of a portion of the dried samples was recorded to determine the amount of graphene. We used 2 mg of the

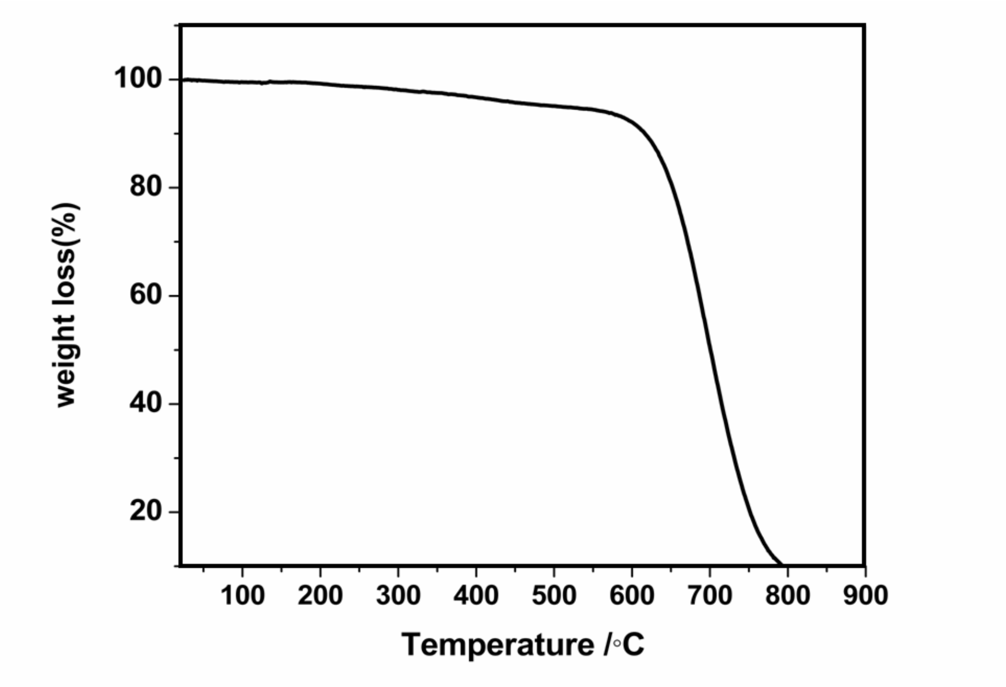


**Figure S2.** TGA thermal curves for the exfoliated graphene under nitrogen atmosphere.

sample and it was heated to 800 $^0$C at a heating rate of 10 $^0$C min$^{-1}$ under nitrogen gas flow at the rate of 40 ml min$^{-1}$. The TGA thermal curve is shown in Fig. S2. From the TGA thermal curve, it is evident that the mass loss occurs in two stages. The mass loss in the first step from 30 $^0$C to 450 $^0$ C corresponds to the decomposition of SDS.[3] There was only a minor loss of 5 % weight for the sample till 650º C. This-clearly showed that the sample contained only a small % (w/w) of the surfactant.

## Synthesis of GO and RGO

Hummers method was followed for the synthesis of GO and RGO.[4] Typically, graphite powder (5 g) was added to concentrated $H_2SO_4$ (98 %, 12 ml) along with $Na_2S_2O_8$ (2.5 g) and $P_2O_5$ (2.5 g). Above mixture was magnetically stirred for 6 h at 80 °C. The solution was centrifuged. The residue was washed with deionized water and dried in vacuum oven. The dried sample obtained above (1 g) was mixed with concentrated $H_2SO_4$ (98%, 23 ml). The reacting mixture was kept in ice bath to maintain the reaction temperature at 0 °C with continuous stirring. $KMnO_4$ (3 g) was slowly added to the solution

while maintaining the temperature at 20 °C. Temperature of the solution was then raised to 35 ºC and maintained for 2 h. Deionised water (45 ml) was added to the solution and the temperature of reaction was raised to 95 °C and stirred for 15 min. Finally, deionized water (200 ml) and $H_2O_2$ (30%, 2.5 ml) were added to terminate the reaction. The solution was sonicated for 1 h to exfoliate and to prepare GO. Finally it was filtered and dried in vacuum oven. GO (10 mg) was sonicated for 2 h in water (50 ml). The sonicated dispersion was centrifuged for 1 h. Upper half of the solution was collected to do further reactions. The above suspension of GO (25 ml) was mixed with water (50 ml), $NH_3$ solution (450 µl, 25%) and hydrazine (80%, 9 µl) to perform the reduction of GO at 95 °C and continued stirring at same temperature for 1 hour. After the completion of the reaction, black coloured RGO was formed.

Characterization of synthesized graphene:

Preliminary characterization of exfoliated graphene dispersed in SDS solution was done by using UV-visible spectroscopy. Typical absorption spectrum of exfoliated graphene is presented in Fig. S3. A strong peak at 214 nm is due to the characteristic absorption of SDS. The UV-Visible spectra reveal that the optical absorption till 350 nm is dominated by the surfactant. The broad absorption beyond 300 nm in the spectrum showing a notifying difference to the blank confirms the optical absorption by graphene.[2] Further analysis of the sample was performed to characterize the morphology of exfoliated graphene.

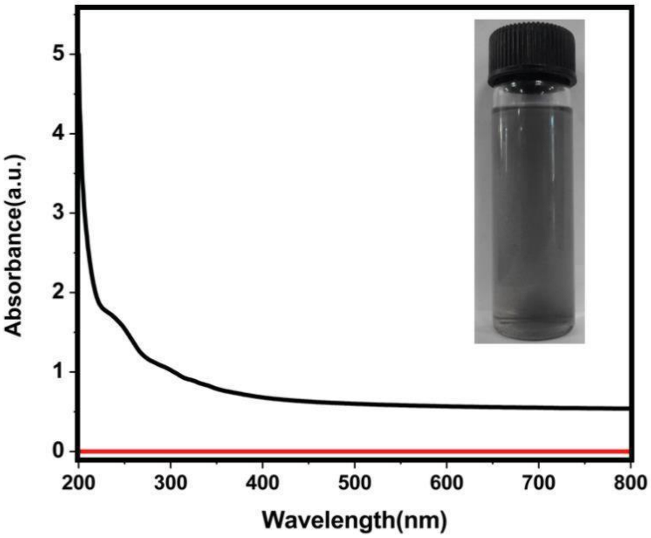


**Figure S3.** UV-Visible absorption spectrum of SDS stabilized graphene dispersion in water. A blank showing absorption of pure water is also shown. Photograph of the graphene dispersion is shown in the inset.

Further moving on to TEM data analysis we observe lots of folded and wrinkled flakes (Fig. S4a). This is a clear evidence of the thinness of exfoliated sheets. High resolution TEM images (Fig. S4b and S4c) showed perfect 2-D crystalline structure of the graphene sheet. Furthermore, the selective area electron diffraction (SAED) patterns were recorded and a typical pattern is shown in Fig. 4d. Presence of two bright concentric rings in the diffraction patterns are due to (1100) and (1120) crystallographic planes of

exfoliated graphene.[5] The concentric rings in the SAED pattern showed subtle difference in their intensity. The inner ring was weaker in intensity than the outer one. This kind of a pattern is known to be due to multilayer graphene.[5] The lateral dimension of majority of the flakes extended upto many micrometres.

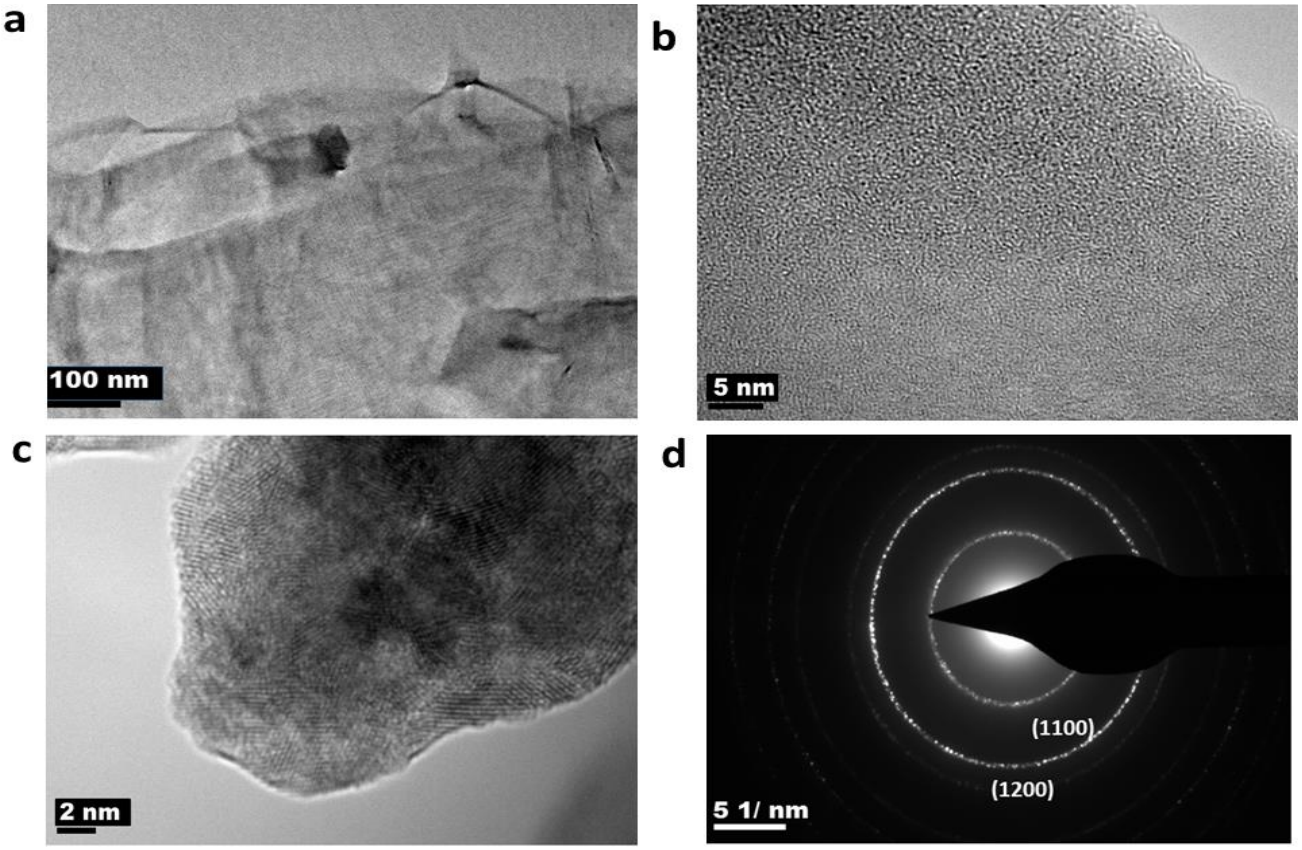


**Figure S4**. TEM images of: (a) a crumbled and folded sheet of graphene (b), (c) HR-TEM images of graphene (d) SAED pattern of graphene sheets.

AFM imaging and analysis of graphene was used to get information about the thickness of graphene sheets. A typical AFM image is shown in Fig. S5a shows the presence of a large sheet of graphene having lateral dimension more than 1 μm along with some smaller flakes. Height profiling data along with images give information about the number of layers present in the synthesized graphene sheets which could be statistically evaluated to get the information about the quality of graphene. A histogram depicting the distribution in thickness of graphene flakes is shown in Fig. S5c conveying the presence of more than 70% of the sheets having stacked layer less than 5. The thinnest flake had (also thickness of ~1 nm. This corresponds to monolayer graphene. However, in case of exfoliated graphene, increased thickness of the sheets could be due to the residual surfactant molecules on the surface.[6] Detailed analysis of large sheets showed that the thickness of the sheets is maximum at the end and minimum in

the middle. This suggested that the graphene sheets were having rolled edges are confirmed proofs about the formation of relatively thin sheets.

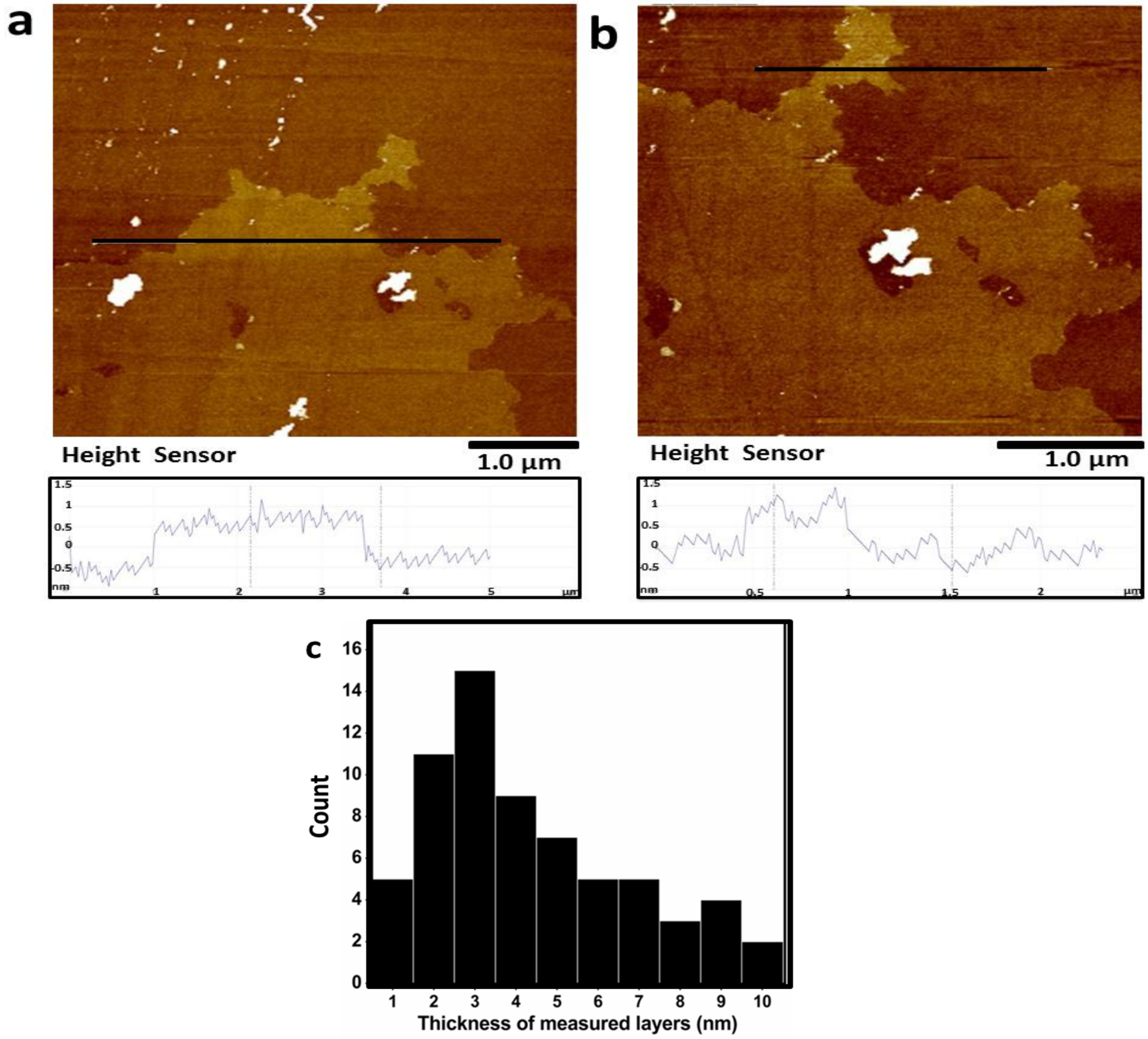


**Figure S5.** (a) and (b) AFM images of Graphene sheets, drop casted on mica surface. Height profile along a line are also shown (c) Height profile statistics derived from analysis of the AFM images of 73 deposited graphene flakes.

XRD analyses of graphite and the exfoliated graphene were done and the diffraction patterns are shown in Fig. S6a. XRD pattern of graphite showed a sharp and strong diffraction peak at $2\theta=26.6$ which was its main characteristic peak corresponding to (200) basal plane along with some small peaks related to (100), (010) (004), (110) planes in graphite. This sharp peak confirmed that the d-spacing in graphite was 3.36Å. [7,8] The exfoliated graphene showed a broad peak at $2\theta=24.8$. The peak shift is due to the

exfoliation of graphite with increase in d spacing from 3.36 to 3.62 Å. This increase in d spacing must be due to insertion of surfactant molecule in between the graphitic layers.

Raman spectroscopy has emerged as a powerful tool to study the quality of graphene. Raman spectra of graphite, exfoliated graphene and RGO are shown in Fig.S6b. All the three materials showed most intense peak a t~1354, ~1625 and ~2700 $cm^{-1}$ corresponding to D, D′ and 2D bands of gra-phene.[9] The D′ band tells us about the graphitic stacking structure.[10] The 2D band was also observed for the three samples at ~2700 $cm^{-1}$. Exfoliated graphene and RGO had an additional peak at ~1354 $cm^{-1}$ corresponding to the D band. The D band correlates to the order/disorder of the graphite edges and was absent for graphite. The presence of D band is expected for RGO because of the residual oxide groups causing defects in sheets. But the appearance of D band in exfoliated graphene does not necessarily confirm the presence of defects. The appearance of D band for exfoliated graphene could be due to the presence of small graphene flakes having lateral dimension less than 1 μm.[6] The size of the laser spot used in Raman spectroscope was larger than 1 μm. Hence, a good deal of small graphene flakes would come under the laser. The smaller flakes will contribute toward the intensity increase of D band as they will be recorded as edge defects.[9] Even, the torn edges within the large graphene flakes as seen in AFM images will also contribute to the intensity of D band. Nevertheless, the intensity of D band for exfoliated graphene was lesser than RGO. Additionally, the degree of apparent disorder can be calculated by the D/D′ intensity ratios.[11 12]

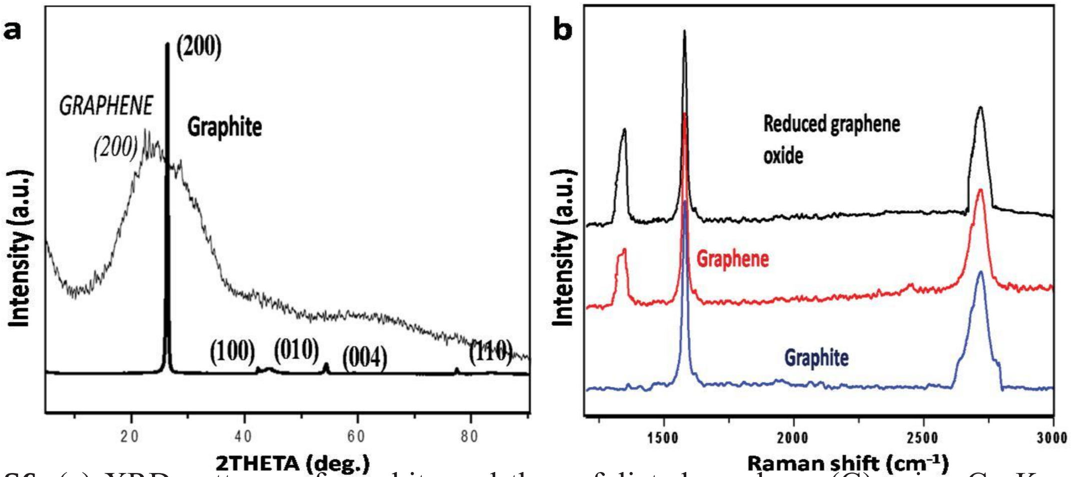


**Figure S6.** (a) XRD patterns of graphite and the exfoliated graphene (G) using Cu Kα radiation (λ= 0.1542 nm, 40 mA, 45 kV) and (b) Raman spectrum of RGO, G and graphite powder.

The calculated D/D′ ratio in case of RGO and G are 0.73 and 0.56 respectively confirming the less defective structure of G sheets than RGO. The 2D band in graphene did not broaden apparently compared to the graphite spectrum. This suggests that the structure of the basal plane in G was still preserved which completely agrees with the SAED results.

## Preparation and characterization of SLCs containing G, RGO and Pd precursor

Transformation of the mixture of the aqueous phase containing surfactant and graphene and the oil phase containing the Pd salt from an opaque emulsion to a transparent and viscous gel gave the primary indication of the formation of hexagonal mesophase. Colour of mesophases; SLC-G1, SLC-RG and SLC-Pd was pale red whereas SLC-G2 was wine red (Figure S7). Colour of the mesophases is due to the typical colour of the $Pd(dba)_2$ complex. The hexagonal mesophases are formed by the self-assembly of infinitely long surfactant–stabilized oil tubes that are regularly arranged in brine solution.[13] The surfactant cylinders have a strong tendency to align and grow parallel to the walls of the container (glass culture tubes here).[13] This growth takes place over a period of few days. Hence, the synthesized mesophases were left undisturbed for few days before further experiments.

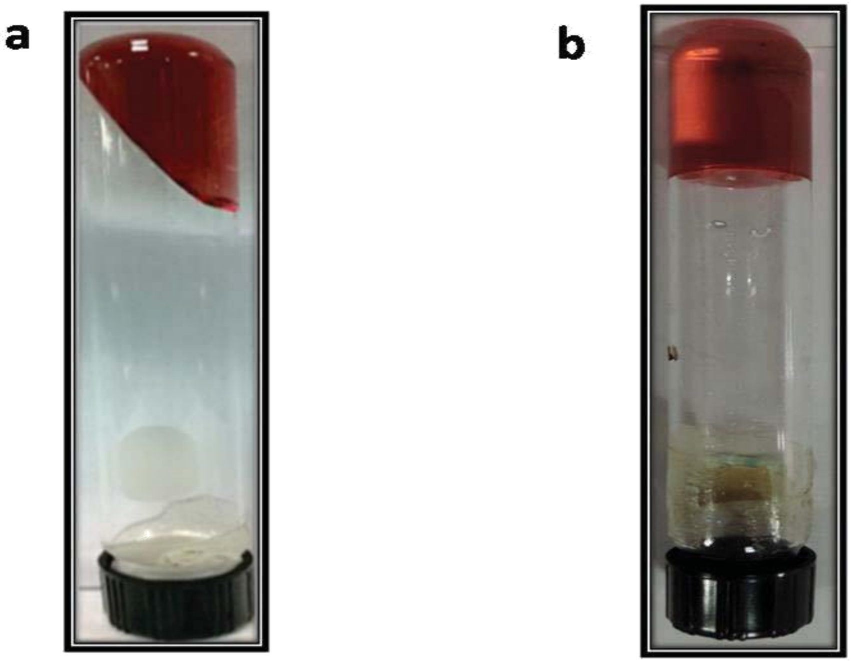


**Figure S7.** Photographs of swollen liquid crystals a) SLC-G1 and b) SLC-G2**.**

The mesophases were further characterized by polarized optical microscopy imaging (POM). A typical POM image of the SLC-G1 is shown in Figure S8. The typical striations like texture of hexagonal mesophases can be seen in this figure. Growth and elongation of the tubes causes evolution of the typical patterns and this is evident in the POM image recorded after 24 h that is shown in Figure S8b. Fan shaped focal conic texture is characteristic of hexagonal mesophases.[14, 15]

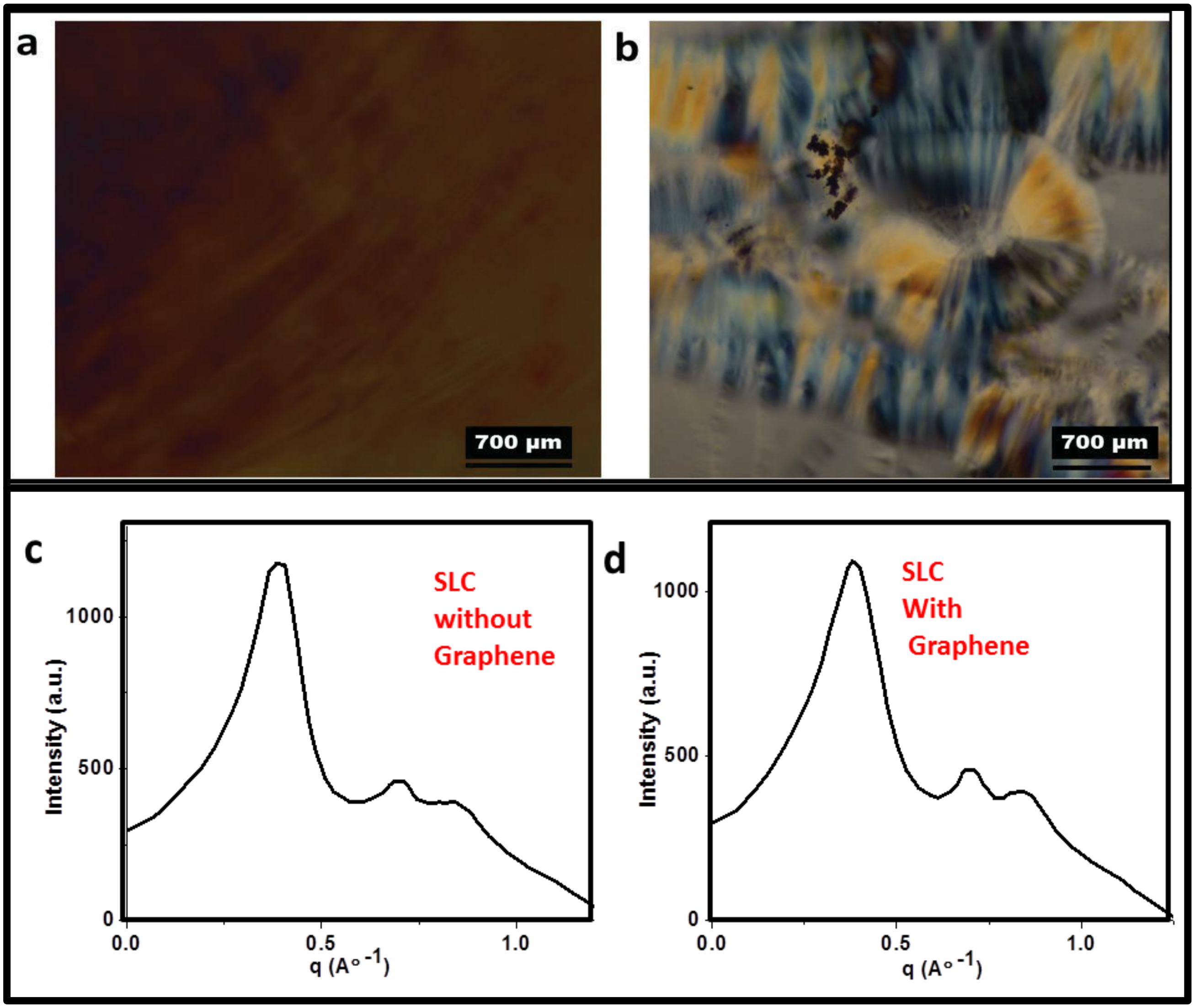


**Figure S8.** Crossed polarized optical microscopy images of a hexagonal mesophase (SLC-G1)(a) immediately after deposition and (b) 24 h after its deposition on a glass slide. SAXS patterns of SLC (c) without graphene (SLC-Pd) and (d) with graphene (SLC-G1).

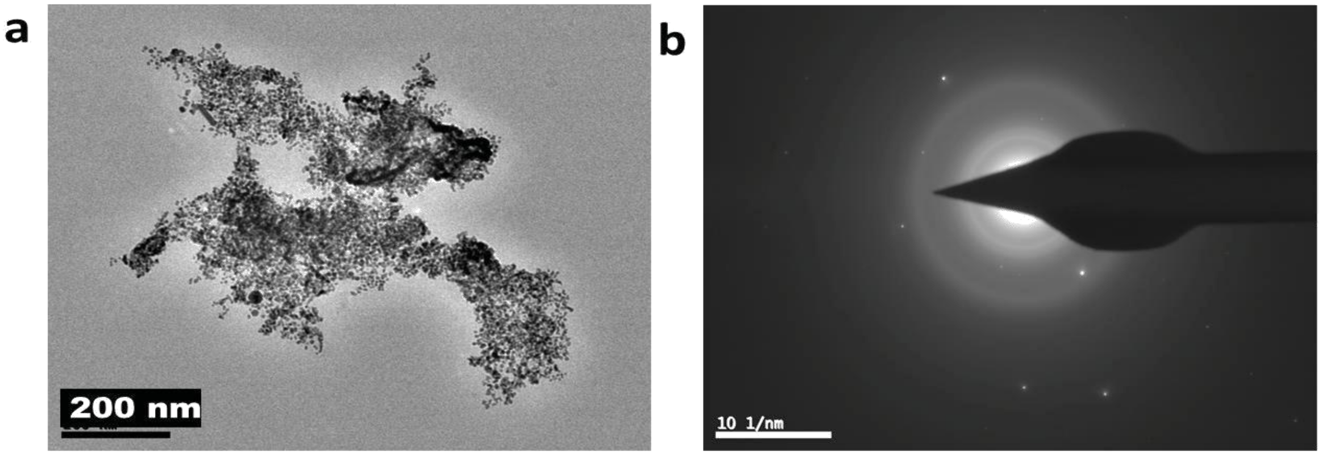


**Figure S9.** Characteristics of palladium nano-particles ($Pd_{0.001M}$) prepared from SLC-Pd (a) TEM image and (b) SAED pattern showing the fcc lattice planes of Pd.

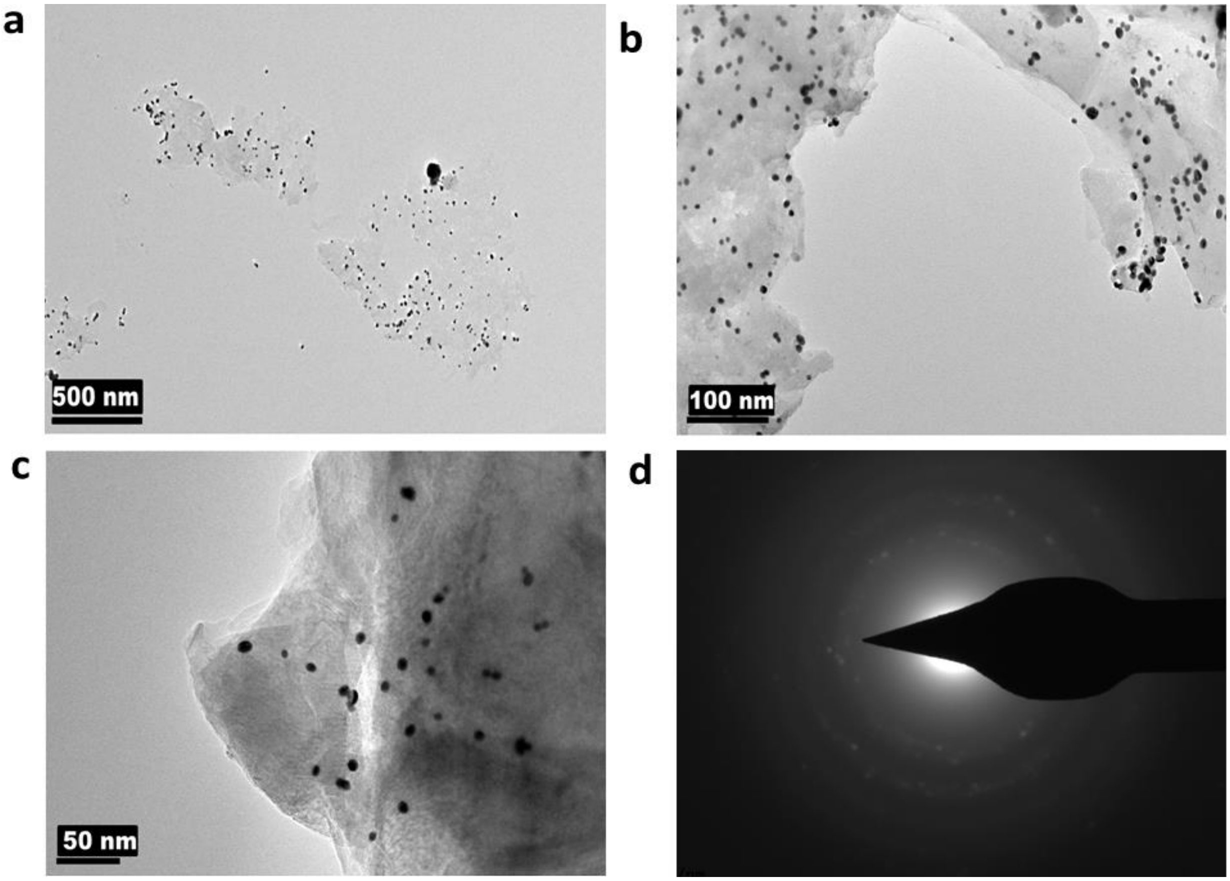


**Figure S10.** Characteristics of palladium/graphene nano-composite ($RGPd_{0.001M}$) prepared from SLC-RG (a), (b), (c)TEM image and (d) SAED pattern.

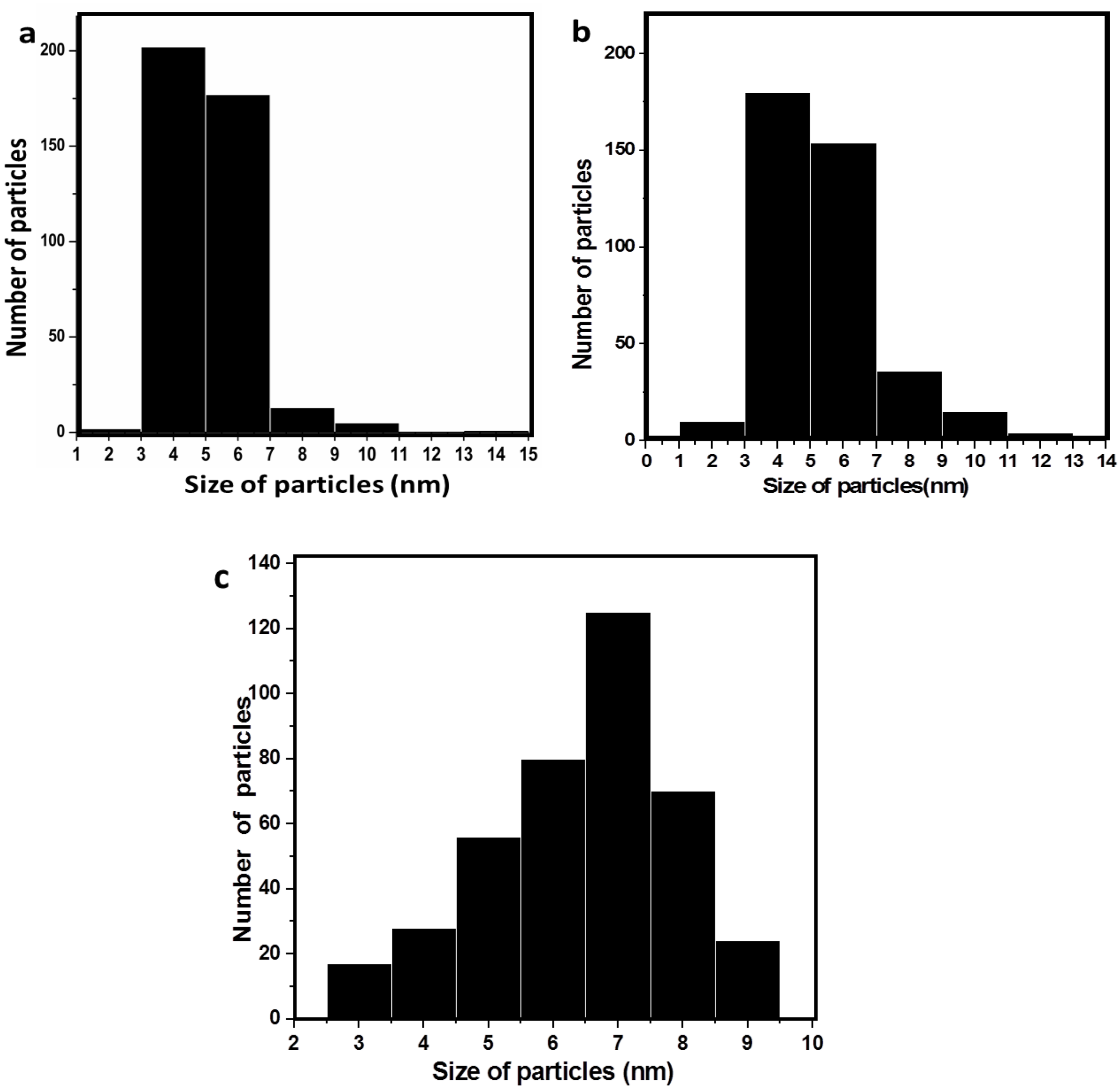


**Figure S 11.** Histograms depicting particle size distributions in (a) $GPd_{0.001M}$, (b) $RGPd_{0.001M}$ and (c) $Pd_{0.001M}$. The particle sizes were measured from TEM images of the respective samples.

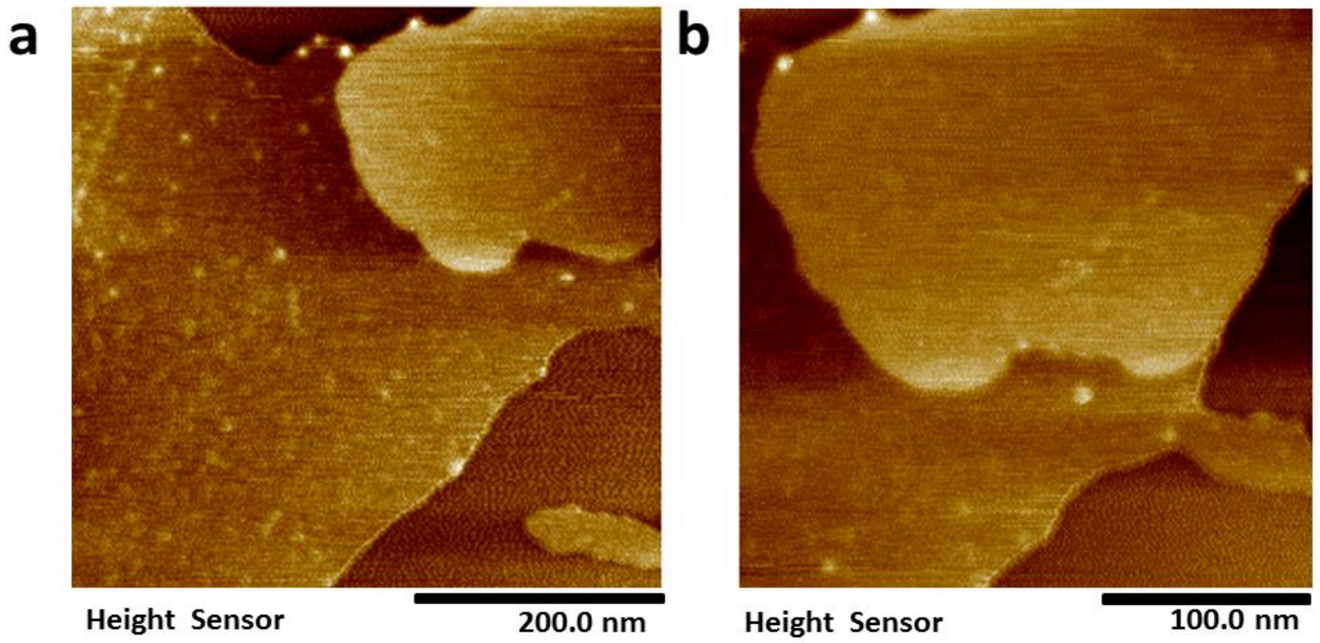


**Figure S12.** AFM images of palladium/graphene nanocomposite ($GPd_{0.001M}$) .

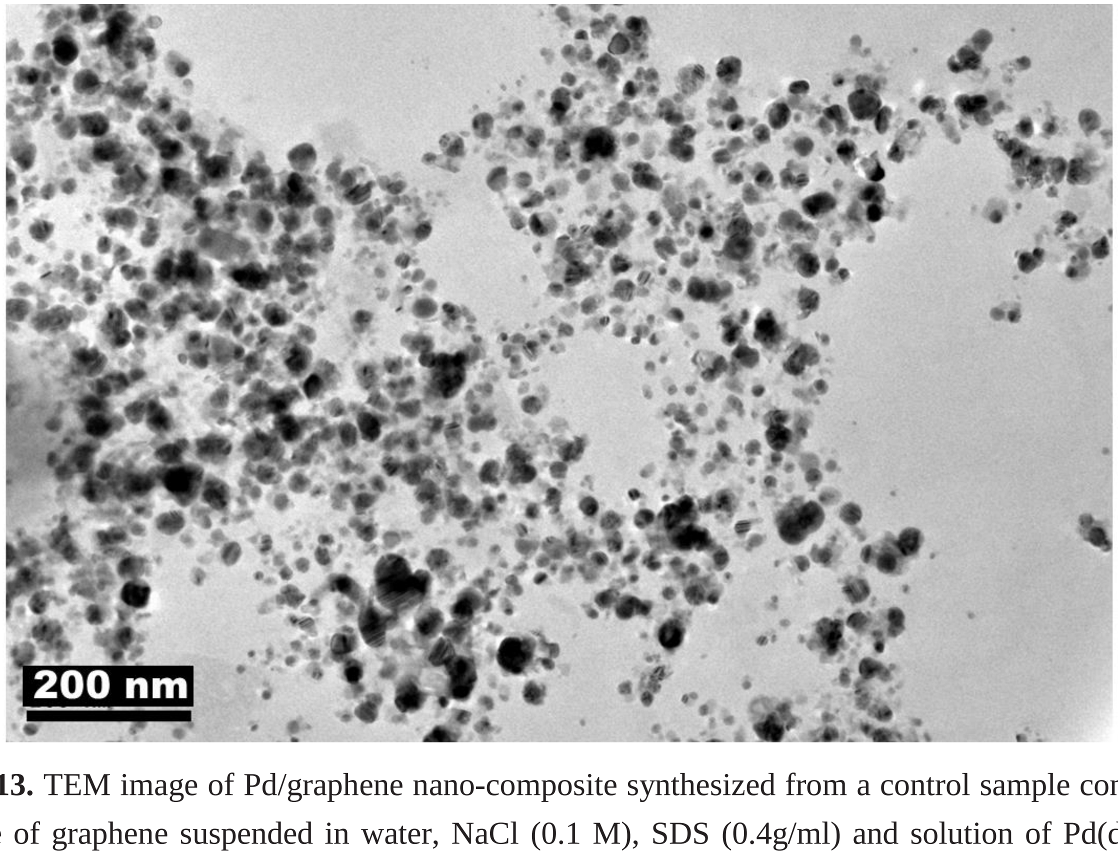


**Figure S13.** TEM image of Pd/graphene nano-composite synthesized from a control sample containing a mixture of graphene suspended in water, NaCl (0.1 M), SDS (0.4g/ml) and solution of $Pd(dba)_2$ in toluene (0.001 M).

a

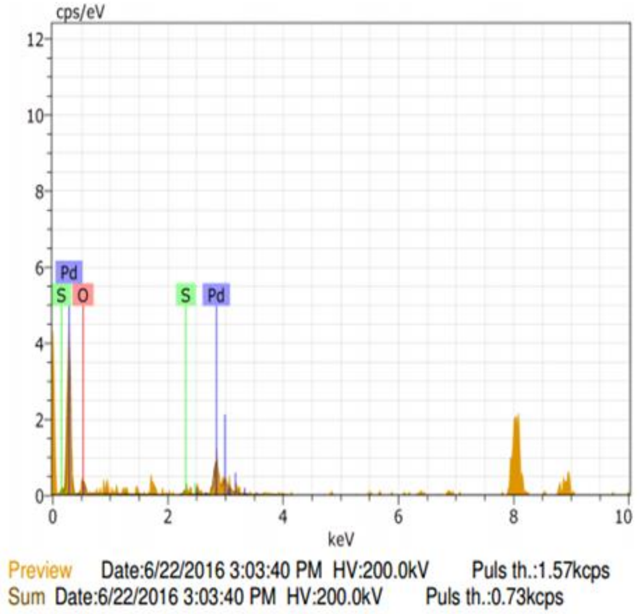


Spectrum: Preview

| Element | Series | Net | unn. C [wt.%] | norm. C [wt.%] | Atom. C [at.%] | Error (3 Sigma) [wt.%] |
|---|---|---|---|---|---|---|
| Oxygen | K-series | 5 | 7.30 | 7.30 | 34.33 | 9.89 |
| Sulfur | K-series | 5 | 0.07 | 0.07 | 0.16 | 0.18 |
| Palladium | K-series | 7 | 92.63 | 92.63 | 65.51 | 113.83 |
| | | Total: | 100.00 | 100.00 | 100.00 | |

b

| S.No | Palladium (%) | Sulfur (%) | Oxygen (%) |
|---|---|---|---|
| 1 | 95 | 0.18 | 4.82 |
| 2 | 93.12 | 1.21 | 5.67 |
| 3 | 92 | 1.03 | 6.97 |
| 4 | 92.45 | 1.09 | 6.45 |
| 5 | 89.12 | 2.01 | 8.87 |
| 6 | 95.2 | 0.03 | 4.77 |
| 7 | 94.36 | 1.11 | 4.53 |
| 8 | 98 | 0.00 | 2 |
| 9 | 93.781 | 0.37 | 5.849 |
| 10 | 92.63 | 1.07 | 6.30 |
| Avg. | 93.566 | 0.801 | 5.6229 |

**Figure S 14. (**a) Typical EDS spectrum of $GPd_{0.001M}$ nanocomposite and (b) Elemental composition of the $GPd_{0.001M}$ nanocomposite that was obtained from EDS spectra those were recorded from 10 different areas of $GPd_{0.001M}$ using EDS analysis.

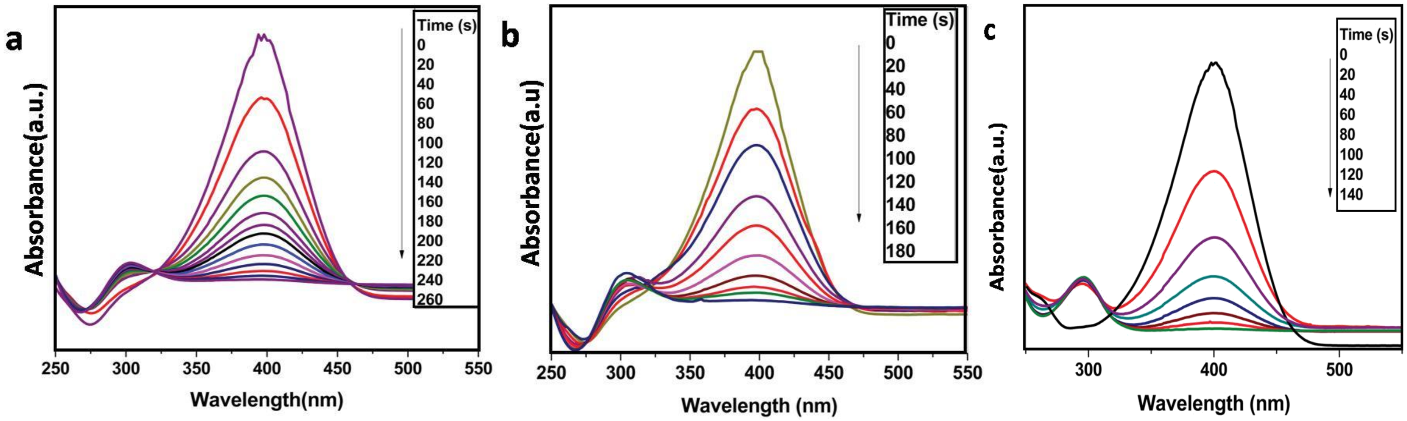


**Figure S15.** Successive UV-visible absorption spectra of p-nitrophenol indicating the progress of hydrogenation by $NaBH_4$ that was catalyzed by: (a) $Pd_{0.001M}$, (b) $GPd_{0.01M}$ and (c) $RGPd_{0.001M}$.

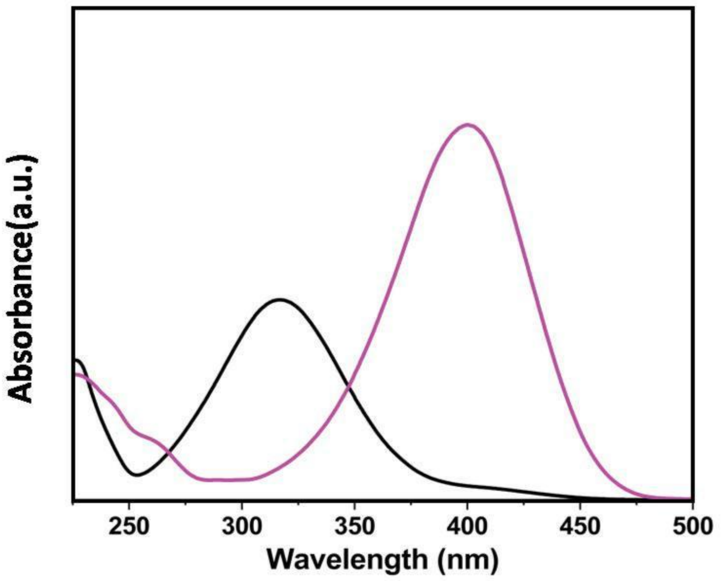


**Figure S16.** (a) UV-vis absorption spectra of p-NP before (i) and after (ii) the addition of $NaBH_4$

## Adsorption of p-NP on $GPd_{0.001}$ and $RGPd_{0.001}$

Adsorption of p-NP on the nanocomposites was studied by using UV-visible absorption spectroscopy, as shown in figure S17. The nanocomposite (20 mg) was added into aqueous solution of p-NP (50 ml, 3 mM). The mixture was thoroughly stirred at room temperature in dark and time depended adsorption experiment was performed. The amount of p-NP adsorbed per unit weight of the nanocomposite, $Q_e$ (mg $g^{-1}$), was calculated from the mass balance equation given below:

$$Q_e = (C_o - C_e)\, Vm^{-1}$$

where $C_o$ is the initial p-NP concentration in the liquid phase (mg $L^{-1}$), $C_e$ is the p-NP concentration at equilibrium (mg $L^{-1}$), V is the volume of p-NP solution used (L), and m is the mass of adsorbent (g). The maximum adsorption capacity ($Q_{max}$) for the nanocomposites $RGPd_{0.001}$ and $GPd_{0.001}$ were 0.066 and 0.089 mg/g respectively.

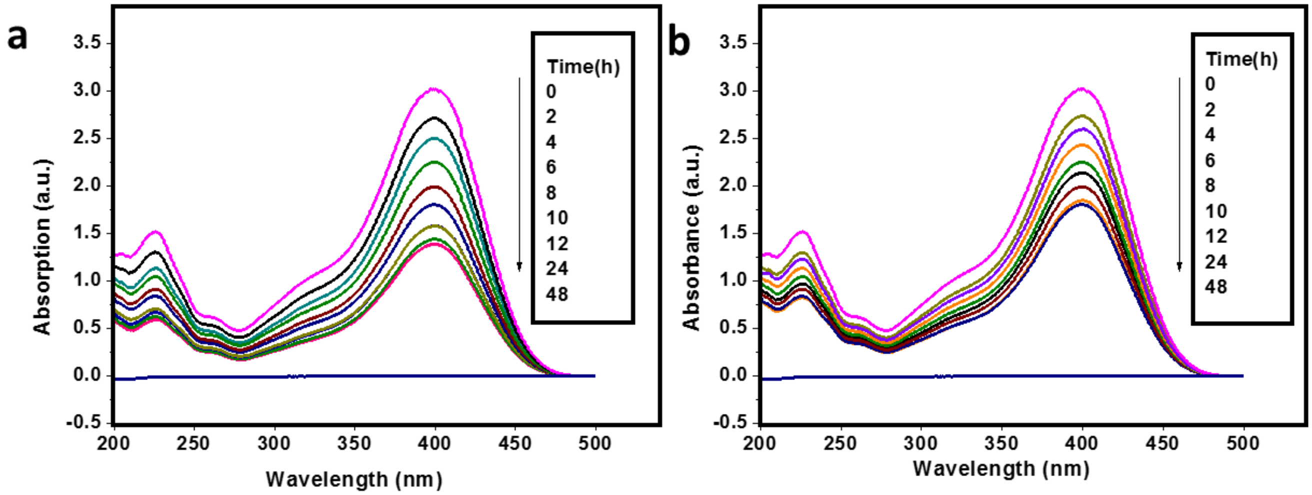


**Figure S17.** Time dependent UV-vis absorption spectra of p-NP showing the adsorption of p-NP on (a) $GPd_{0.001M}$ and (b) $RGPd_{0.001M}$.

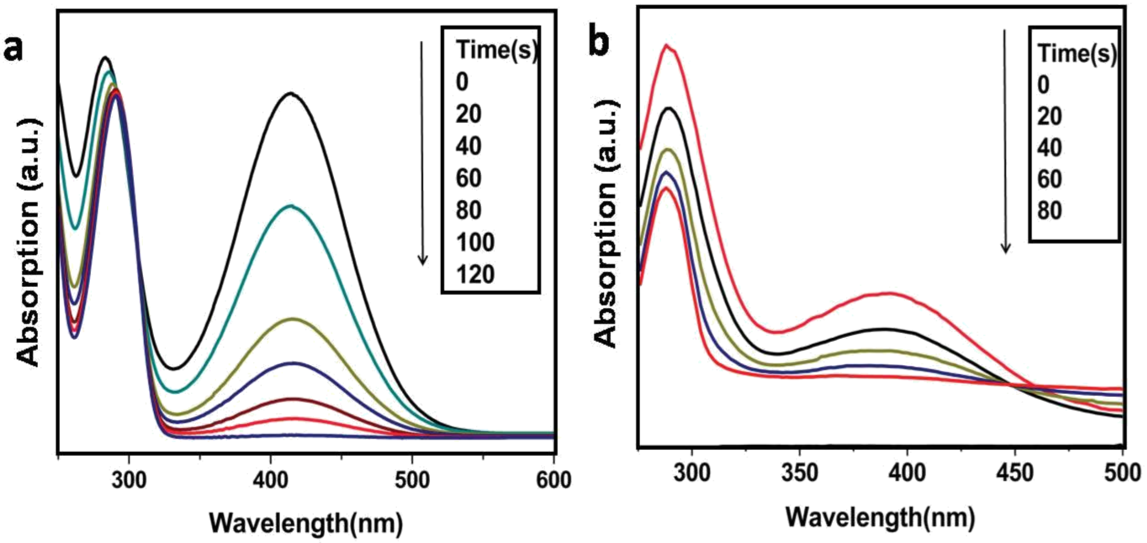


**Figure S18:** Evolution of the UV-vis absorption spectra with time, showing the efficient catalytic activity of $GPd_{0.001M}$ in the borohydride reduction of: (a) o-nitrophenol and (b) m-nitrophenol.

| S.No. | Catalyst | Diameter (nm) | Rate Constant, k ($s^{-1}$) | Concentration of Catalyst (mg/ml) | References |
|---|---|---|---|---|---|
| 1. | $Pd_{0.001M}$ | 7.7 ± 1.7 | 0.008 | 0.075 | This work |
| 2. | $RGPd_{0.001M}$ | 4.7 ± 1.4 | 0.059 | 0.075 | This work |
| 3. | $GPd_{0.01M}$ | 10.32 | 0.015 | 0.075 | This work |
| 4. | $GPd_{0.001M}$ | 4.2 ± 1.6 | 0.133 | 0.075 | This work |
| 5. | $Pd@Ni_xB\text{-}SiO_2$/RGO | 2.5-4 | 0.017 | 2.5 | [16] |
| 6. | Pd/GO | 4.3 | $34.3 \times 10^{-3}$ | 0.25 | [17] |
| 7. | Pd/C | 3.4 | $8.83 \times 10^{-3}$ | NA | [18] |
| 8. | Pd/microgel-PS | 3.8 | $1.5 \times 10^{-3}$ | NA | [19] |
| 9. | Template-free | 4-5 | 0.000133 | 0.17 | [20] |
| 10. | Pd-RGO | 10-20 | $2.35\times10^{-3}$ | 0.5 | [21] |
| 11. | $Pd_{0.05}$/RGO | 6.6 | 0.036 | 0.093 | [22] |

**Table S1.** Comparison of rate constants of synthesized Pd nano-catalysts loaded on different supports for the reduction of 4-nitrophenol.

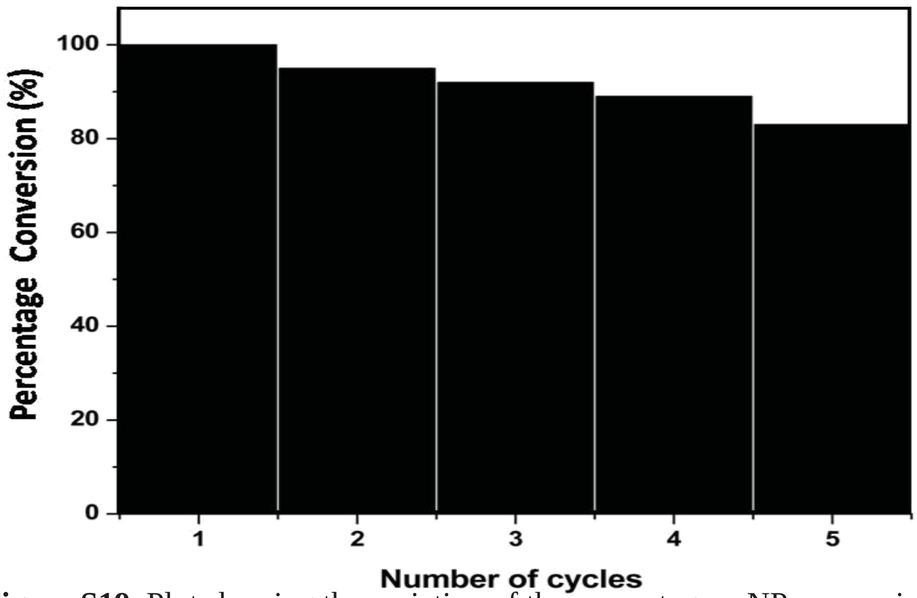


**Figure S19.** Plot showing the variation of the percentage p-NP conversion at 120 s after successive recycling of the catalyst, $GPd_{0.001M}$.

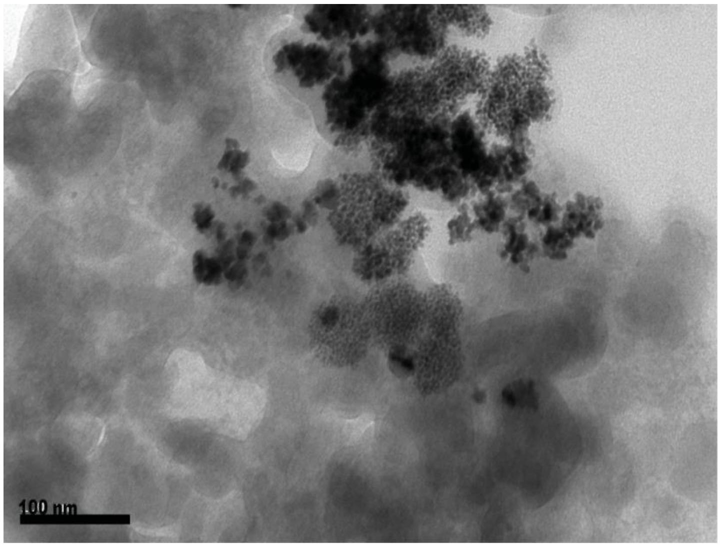


**Figure S20**. A representative TEM image of $GPd_{0.001M}$ collected after the $5^{th}$ cycle of the hydrogenation reaction showing the continued attachment of Pd nanoparticles on graphene sheets**.**

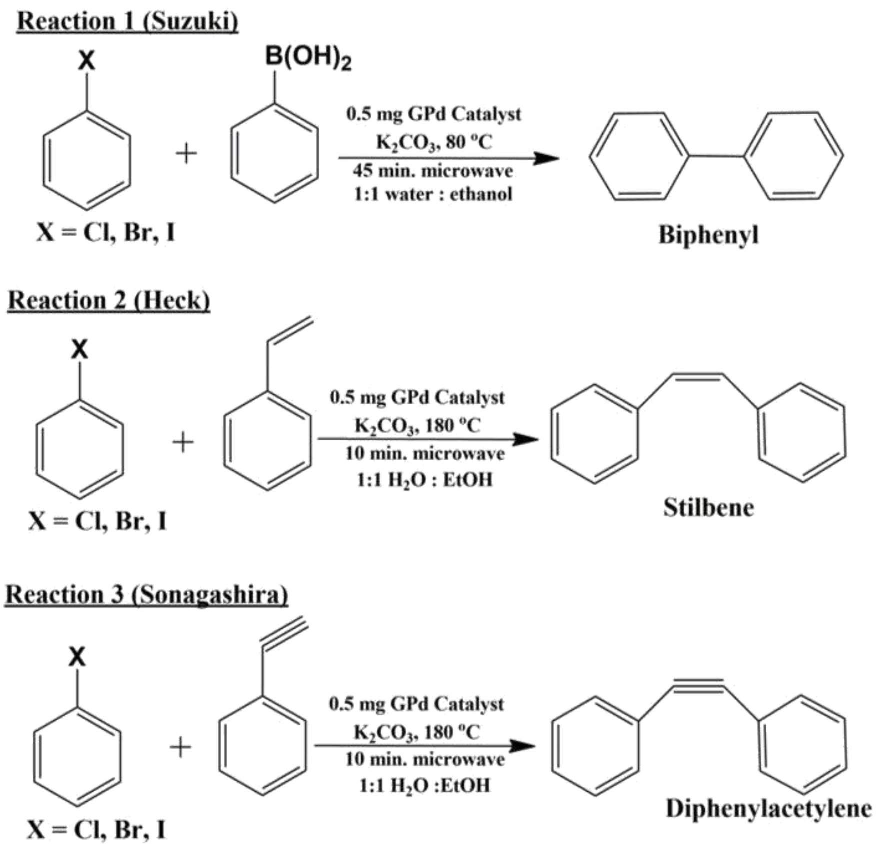


**Scheme S1:** Different reaction schemes of Carbon−Carbon Cross Coupling Reactions.

***Optimization of base and solvents***

The catalytic activities of the four different catalysts ($GPd_{0.001}$, $GPd_{0.01}$, $RGPd_{0.001}$ and $Pd_{0.001}$) were tested initially in the Suzuki reaction using Chloro benzene and phenyl boronic acid as reactants. $GPd_{0.001\ M}$ was found to be the best catalyst. Then, the effect of base on the efficiency of $GPd_{0.001\ M}$ was tested using five different bases: NaOH, $K_2CO_3$, $Na_2CO_3$, $Na_2PO_4$ and $(C_2H_5)_3N$. The results are given in Table S2. The effect of solvent was also studied using four different solvents such as water, ethanol, toluene and water ethanol 1:1 mixture in Suzuki reaction using chloro benzene and the results are given in Table S3.

| **Base** | **Reaction** | **Yield %** |
|---|---|---|
| NaOH | Suzuki | 72 |
| $K_2CO_3$ | Suzuki | 83 |
| $Na_2CO_3$ | Suzuki | 74 |
| $Na_2PO_4$ | Suzuki | 73 |
| $(C_2H_5)_3N$ | Suzuki | 68 |

**Table S2.** Effect of different bases in Suzuki Carbon−Carbon cross coupling reactions between bromobenzene and phenyl boronic acid under microwave irradiation.

| Solvent | Yield % |
|---|---|
| Water | 75 |
| Ethanol | 78 |
| Toluene | 62 |
| Water+ Ethanol | 83 |

**Table S3.** Effect of different solvents in Suzuki Carbon−Carbon Cross Coupling Reactions between bromobenzene and phenyl boronic acid under microwave irradiation in presence of $K_2CO_3$**.**

| Scheme | Reaction | Reactant | Product | Yield (%) | Experimental conditions. |
|---|---|---|---|---|---|
| S1 | Suzuki | Chloro Benzene, phenyl boronic acid | Biphenyl | 83 | 45 min, 90°C, 0.5 mg $GPd_{0.001}$, 2 ml water and 2 ml ethanol, $K_2CO_3$. In microwave under sealed condition. |
| S2 | Suzuki | Bromo Benzene, phenyl boronic acid | Biphenyl | 86 | |
| S3 | Suzuki | Iodo Benzene, phenyl boronic acid | Biphenyl | 87 | |
| H1 | Heck | Chloro Benzene, Styrene | Stilbene | 81 | 10 min, 180 °C, 0.5 mg $GPd_{0.001}$, 2 ml water and 2 ml ethanol, $K_2CO_3$ base, in microwave under sealed conditions. |
| H2 | Heck | Bromo Benzene, Styrene | Stilbene | 85 | |
| H3 | Heck | Iodo Benzene, Styrene | Stilbene | 93 | |
| SG1 | Sonogashira | Chloro Benzene, phenylacetalene | Biphenyl | 83 | 10 min, 180°C, 0.5 mg $GPd_{0.001}$, 2 ml water and 2 ml ethanol, $K_2CO_3$ base, in microwave under sealed conditions. |
| SG2 | Sonogashira | Bromo Benzene, phenylacetalene | Biphenyl | 87 | |
| SG3 | Sonogashira | Iodo Benzene, phenylacetalene | Biphenyl | 95 | |

**Table S4**. Catalytic activity of $GPd_{0.001}$ in various Carbon−Carbon Cross Coupling reactions under microwave irradiation.

**NMR**

**Sonagoshira**

$^{1}$H NMR ($CDCl_3$500 MHz) δ 7.54 – 7.52 (m, 4H) δ 7.36 -7.33 (m, 6H);

$^{13}$C NMR ($CDCl_3$ 500 MHz) δ 131.58, δ128.3, δ 128.2, δ 123.24, δ 89.34;

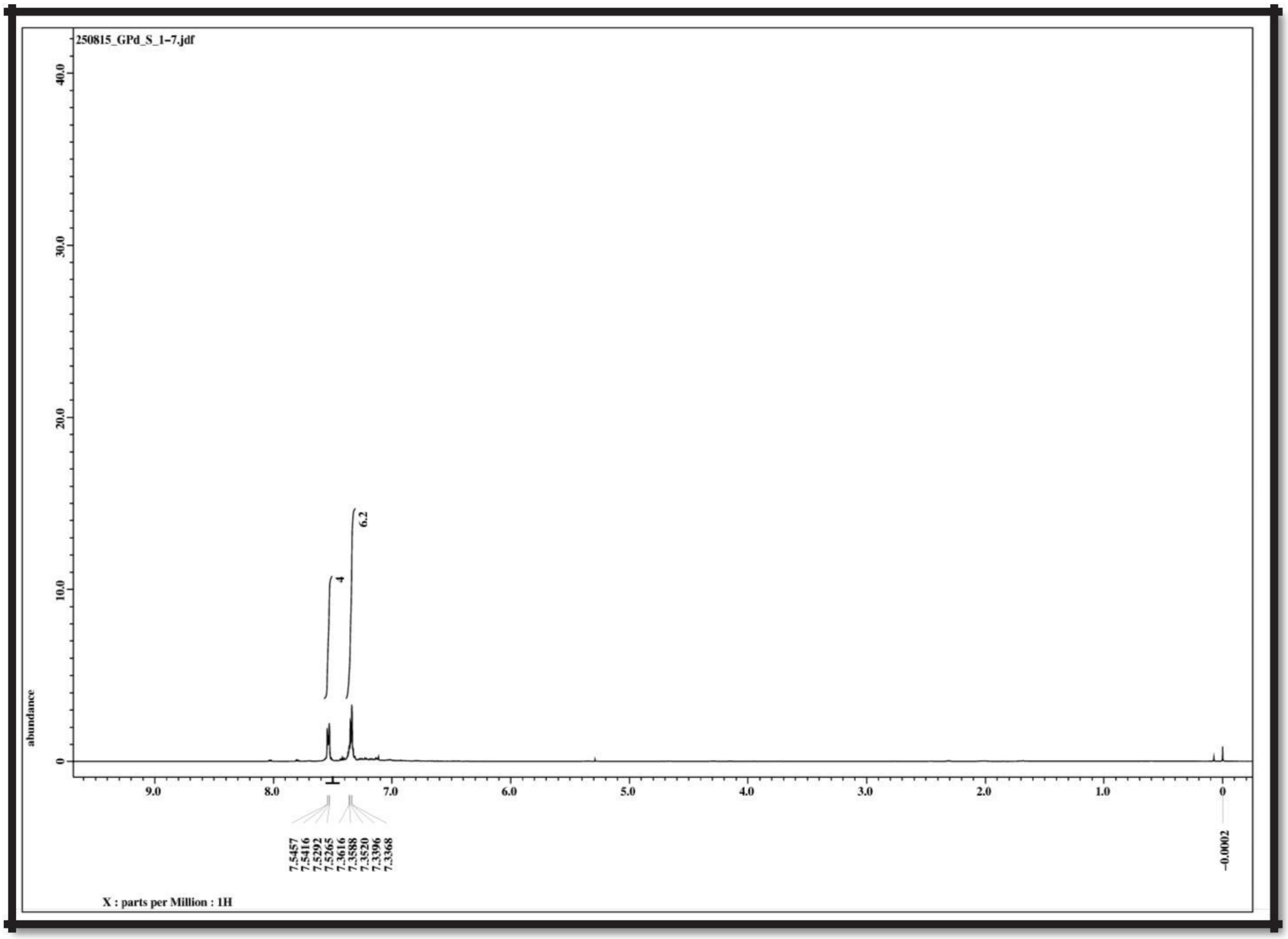


**Figure S 21** $^{1}$H NMR spectra of product formed in Sonogashira reactions product Stillbene**.**

**Suzuki**

$^{1}$H NMR ($CDCl_3$ 500 MHz) δ 7.60-7.58 (m, 4H), δ 7.45--7.42 (m, 4H), δ 7.36-7.33 (m, 2H);

$^{13}$C NMR ($CDCl_3$ 500 MHz) δ 141.21, 128.73, 127.22, 127.15;

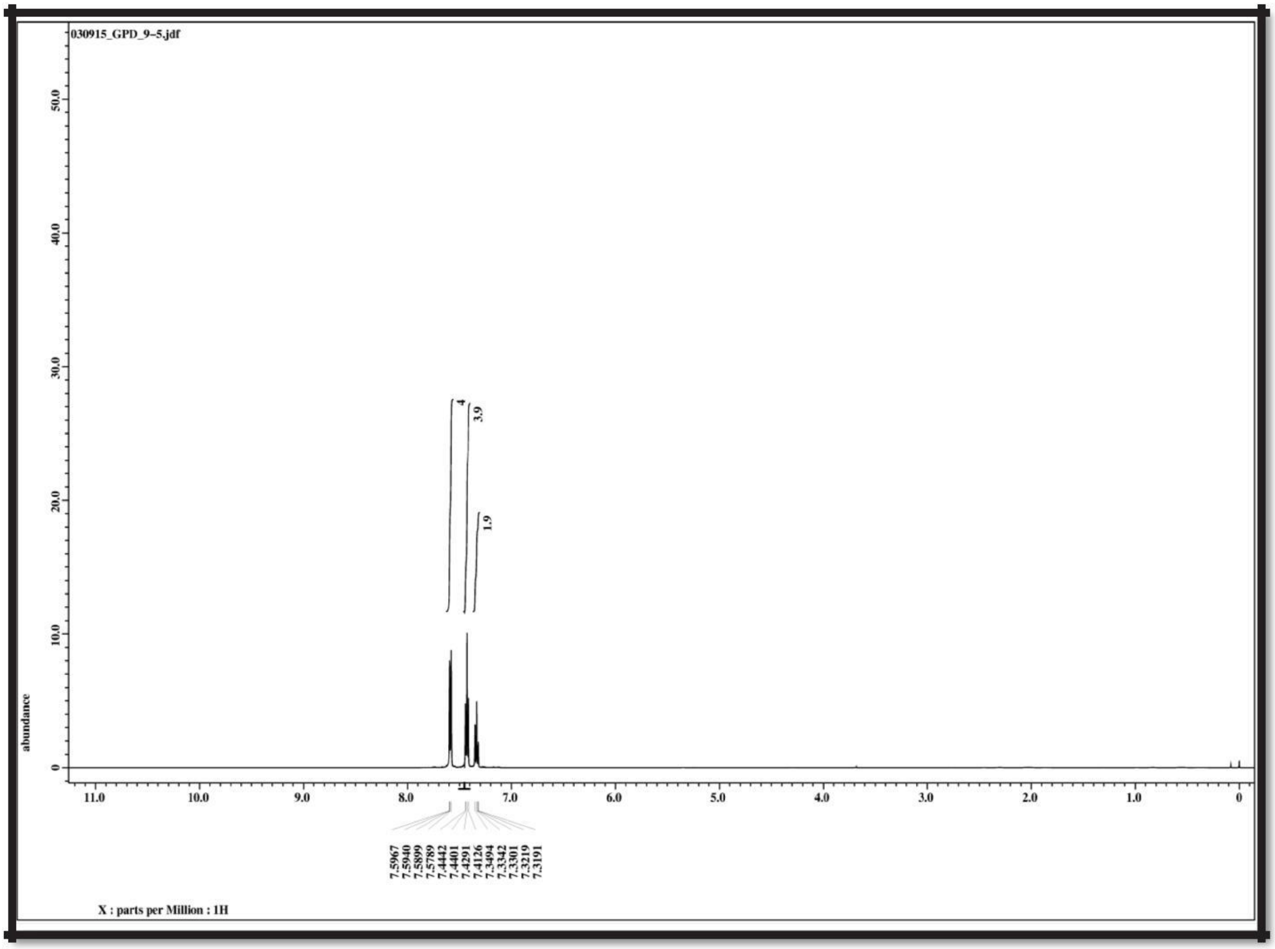


**Figure S22.** $^{1}$H NMR spectra of the product formed (biphenyl) in Suzuki-Miyaura coupling reactions

## Heck

$^{1}H$ NMR ($CDCl_3$ 500 MHz) δ 7.52-7.50 (m, 4H), δ 7.37--7.32 (m, 4H), δ 7.26-7.23 (m, 2H), δ 7.10 (2H);

$^{13}C$ NMR ($CDCl_3$ 500 MHz) δ = 137.29, 128.65, 127.60, 127.21;

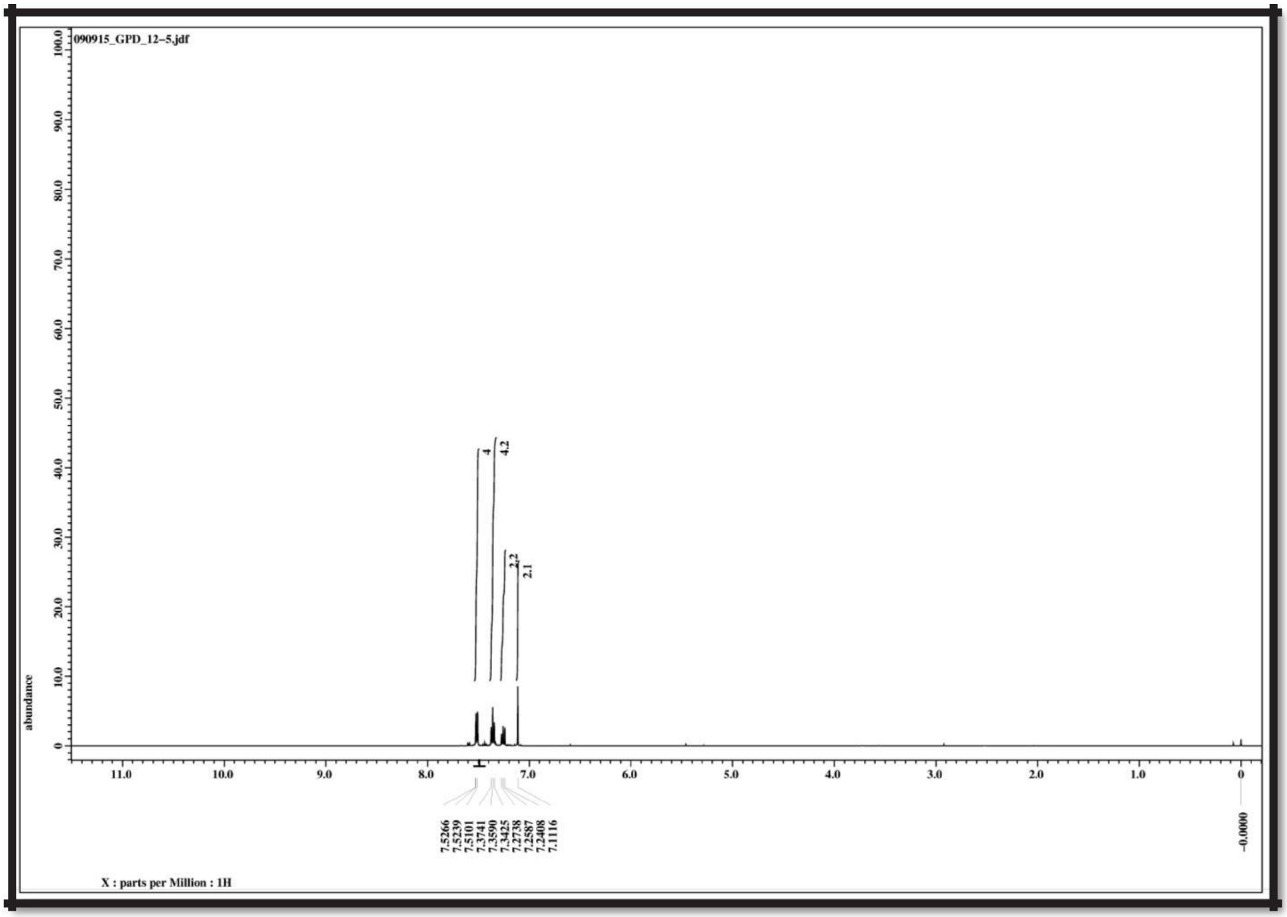


**Figure S23.** $^{1}H$ NMR spectra of product formed in Heck Reactions styrene.

| S. No. | Catalyst /support (Concentration) | Particle size (nm) | Reactant (Concentration) | T (°C) | (solvent) | Duration | Base | Yield (%) | Ref. |
|---|---|---|---|---|---|---|---|---|---|
| 1. | Pd/RGO (0.48 mol%) | 5-7 | $C_6H_5Br$ (1 mmol) | 80 | $H_2O$ | 0.5 h | $K_2CO_3$ | 95 | 23 |
| 2. | Pd-GO (0.25 mol%) | 4 | $C_6H_5Br$ (0.5 mmol) | 80 | $H_2O$/Et hanol | 4 h | $Na_2CO_3$ | 100 | 24 |
| 3. | ERGO-Pd (2 mg) | 6 | $C_6H_5I$ (1 mmol) | reflux | Ethanol | 1 h | $K_2CO_3$ | 95 | 25 |
| 4. | Pd/G (10 mg) | 4-8 | $C_6H_5I$ (1 mmol) | 50 | Ethanol | 10 min | $K_3PO_4$ | 97 | 26 |
| 5. | Pd2+/GO (1 mol%) | 7 | 4-$CF_3$ substituted $C_6H_5Cl$ (0.5 mmol) | 80 | DMA/ $H_2O$ | 24 h | $Na_2CO_3$ | 100 | 27 |
| 6 | Pd2+/GO (0.25 mol%) | 7 | $C_6H_5I$ (0.51 mmol) | 80 | PrOH/ $H_2O$ | 24 h | $Na_3PO_4$ | 95 | 27 |
| 7 | Pd/GO (2 mol%) | 2 | $C_6H_5Br$ (0.51 mmol) | 80 | Ethanol /$H_2O$ | 5 h | $K_3PO_4$ | 91 | 28 |
| 8 | Pd-Co/G (0.02 mmol%) | 9 | $C_6H_5I$ (0.51 mmol) | 80 | Ethanol /$H_2O$ | 2 h | $Na_2Co_3$ | 76 | 29 |
| 9 | Pd-Co/G (0.02 mmol%) | 9 | $C_6H_5I$ (1 mmol) | 80 | THF/$H_2$ O | 12 h | $Et_3N$ | 98 | 29 |
| 10 | Pd/G (0.3 mol%) | 7-9 | $C_6H_5Br$ (0.32 mmol) | 80 (Micro wave) | $H_2O$/Et hanol | 5 min | $K_2CO_3$ | 100 | 30 |
| 11 | Pd/PRGO (0.5 mol%) | 8 | $C_6H_5Br$ (0.32 mmol) | 120 | $H_2O$/Et hanol | 45 min | $K_2CO_3$ | 100 | 31 |
| 12 | $GPd_{0.001M}$ (0.5 mg / 0.33 mmol %) | 4.2 | $C_6H_5Cl$ (0.48 mmol) | 90 (micro wave) | $H_2O$/Et hanol | 45 min | $K_2CO_3$ | 87 | This work |
| 13 | $RGPd_{0.001M}$ (0.5 mg / 0.35 mmol %) | 4.7 | $C_6H_5Cl$ (0.48 mmol) | 90 (Micro wave) | $H_2O$/Et hanol | 45 min | $K_2CO_3$ | 78 | This work |

ERGO-Pd: Electrochemically co-deposited Pd nanoparticles and reduced graphene oxide ; Pd/G: Pd nanoparticles dispersed on Graphene; DBU: 1,8-diazabicyclo[5.4.0]undecene; Pd2+/GO: $Pd^{2+}$ -Exchanged graphite oxide; DMA: N,N-dimethylacetamide; PrOH: Propanol; PRGO:partially reduced graphene oxide nanosheet.

**Table S5.** Comparison of the catalytic activities of the synthesized G/Pd nanocomposites with other Pd-graphene nanocomposites for Suzuki-Miyaura coupling reaction.

# References


1 Lotya, M., King, P. J., Khan, U., De, S. & Coleman, J. N. High-concentration, surfactant-stabilized graphene dispersions. *ACS nano* 4, 3155-3162 (2010).

2 Hernandez, Y. *et al.* High-yield production of graphene by liquid-phase exfoliation of graphite. *Nature nanotechnology* 3, 563-568 (2008).

3 Ramimoghadam, D., Hussein, M. Z. B. & Taufiq-Yap, Y. H. The effect of sodium dodecyl sulfate (SDS) and cetyltrimethylammonium bromide (CTAB) on the properties of ZnO synthesized by hydrothermal method. *International journal of molecular sciences* 13, 13275-13293 (2012).

4 William, S., Hummers, J. & Offeman, R. Preparation of graphitic oxide. *J Am Chem Soc* 80, 1339 (1958).

5 Wang, H. *et al.* Water-soluble polymer exfoliated graphene: as catalyst support and sensor. *The Journal of Physical Chemistry B* 117, 5606-5613 (2013).

6 Vadukumpully, S., Paul, J. & Valiyaveettil, S. Cationic surfactant mediated exfoliation of graphite into graphene flakes. *Carbon* 47, 3288-3294 (2009).

7 Li, Z., Lu, C., Xia, Z., Zhou, Y. & Luo, Z. X-ray diffraction patterns of graphite and turbostratic carbon. *Carbon* 45, 1686-1695 (2007).

8 Fang, M., Wang, K., Lu, H., Yang, Y. & Nutt, S. Single-layer graphene nanosheets with controlled grafting of polymer chains. *Journal of Materials Chemistry* 20, 1982-1992 (2010).

9 Casiraghi, C., Pisana, S., Novoselov, K., Geim, A. & Ferrari, A. Raman fingerprint of charged impurities in graphene. *Applied Physics Letters* 91, 233108 (2007).

10 Qian, W. *et al.* Solvothermal-assisted exfoliation process to produce graphene with high yield and high quality. *Nano Research* 2, 706-712 (2009).

11 Cançado, L. G. *et al.* Quantifying defects in graphene via Raman spectroscopy at different excitation energies. *Nano letters* 11, 3190-3196 (2011).

12 Calizo, I., Balandin, A., Bao, W., Miao, F. & Lau, C. Temperature dependence of the Raman spectra of graphene and graphene multilayers. *Nano letters* 7, 2645-2649 (2007).

13 Ramos, L. & Fabre, P. Swelling of a lyotropic hexagonal phase by monitoring the radius of the cylinders, Langmuir, 13, 682-686 (1997).

14 Dutt, S., Siril, P. F., Sharma, V & Periasamy, S Gold$_{core}$–polyaniline$_{shell}$ composite nanowires as a substrate for surface enhanced Raman scattering and catalyst for dye reduction, New Journal of Chemistry, 39 (2), 902-908, (2015).

15 Dutt, S & Siril, P. F. Morphology controlled synthesis of polyaniline nanostructures using swollen liquid crystal templates, Journal of Applied Polymer Science 131 (18), 40800-40808 (2014).

16 Krishna, R. *et al.* Reduction of 4-nitrophenol to 4-aminophenol using a novel Pd@ Ni x B– SiO 2/RGO nanocomposite: enhanced hydrogen spillover and high catalytic performance. *RSC Advances* 5, 60658-60666 (2015).

17 Sun, W. *et al.* Fabrication of highly dispersed palladium/graphene oxide nanocomposites and their catalytic properties for efficient hydrogenation of p-nitrophenol and hydrogen generation. *International Journal of Hydrogen Energy* 39, 9080-9086 (2014).

18 Fang, Y. & Wang, E. Simple and direct synthesis of oxygenous carbon supported palladium nanoparticles with high catalytic activity. *Nanoscale* 5, 1843-1848 (2013).

19 Mei, Y., Lu, Y., Polzer, F., Ballauff, M. & Drechsler, M. Catalytic activity of palladium nanoparticles encapsulated in spherical polyelectrolyte brushes and core-shell microgels. *Chemistry of Materials* 19, 1062-1069 (2007).

20 Halder, A., Patra, S., Viswanath, B., Munichandraiah, N. & Ravishankar, N. Porous, catalytically active palladium nanostructures by tuning nanoparticle interactions in an organic medium. *Nanoscale* 3, 725-730 (2011).

21 Wang, Z., Xu, C., Gao, G. & Li, X. Facile synthesis of well-dispersed Pd–graphene nanohybrids and their catalytic properties in 4-nitrophenol reduction. *RSC Advances* 4, 13644-13651 (2014).
22 Sun, J., Fu, Y., He, G., Sun, X. & Wang, X. Catalytic hydrogenation of nitrophenols and nitrotoluenes over a palladium/graphene nanocomposite. *Catalysis Science & Technology* 4, 1742-1748 (2014).
23 Hoseini, S. J., Dehghani, M. & Nasrabadi, H. Thin film formation of Pd/reduced-graphene oxide and Pd nanoparticles at oil–water interface, suitable as effective catalyst for Suzuki–Miyaura reaction in water. *Catalysis Science & Technology* 4, 1078-1083 (2014).
24 Scheuermann, G. M., Rumi, L., Steurer, P., Bannwarth, W. & Mülhaupt, R. Palladium nanoparticles on graphite oxide and its functionalized graphene derivatives as highly active catalysts for the Suzuki− Miyaura coupling reaction. *Journal of the American Chemical Society* 131, 8262-8270 (2009).
25 Shendage, S. S., Singh, A. S. & Nagarkar, J. M. Facile approach to the electrochemical synthesis of palladium-reduced graphene oxide and its application for Suzuki coupling reaction. *Tetrahedron Letters* 55, 857-860 (2014).
26 Li, N. *et al.* Graphene-Pd composite as highly active catalyst for the Suzuki-Miyaura coupling reaction. *Journal of Nanoscience and Nanotechnology* 10, 6748-6751 (2010).
27 Rumi, L., Scheuermann, G. M., Muelhaupt, R. & Bannwarth, W. Palladium Nanoparticles on Graphite Oxide as Catalyst for Suzuki-Miyaura, Mizoroki-Heck, and Sonogashira Reactions. *Helvetica Chimica Acta* 94, 966-976 (2011).
28 Ioni, Y. V. *et al.* Activity of palladium nanoparticles on graphene oxide in the Suzuki—Miyaura reaction. *Russian Chemical Bulletin* 61, 1825-1827 (2012).
29 Feng, Y.-S., Lin, X.-Y., Hao, J. & Xu, H.-J. Pd–Co bimetallic nanoparticles supported on graphene as a highly active catalyst for Suzuki–Miyaura and Sonogashira cross-coupling reactions. *Tetrahedron* 70, 5249-5253 (2014).
30 Siamaki, A. R., Abd El Rahman, S. K., Abdelsayed, V., El-Shall, M. S. & Gupton, B. F. Microwave-assisted synthesis of palladium nanoparticles supported on graphene: A highly active and recyclable catalyst for carbon–carbon cross-coupling reactions. *Journal of catalysis* 279, 1-11 (2011).
31 Moussa, S., Siamaki, A. R., Gupton, B. F. & El-Shall, M. S. Pd-partially reduced graphene oxide catalysts (Pd/PRGO): laser synthesis of Pd nanoparticles supported on PRGO nanosheets for carbon–carbon cross coupling reactions. *Acs Catalysis* 2, 145-154 (2011).
